# *o*-Vanillin Derived Schiff Bases and Their Organotin(IV) Compounds: Synthesis, Structural Characterisation, In-Silico Studies and Cytotoxicity

**DOI:** 10.3390/ijms20040854

**Published:** 2019-02-15

**Authors:** Enis Nadia Md Yusof, Muhammad A. M. Latif, Mohamed I. M. Tahir, Jennette A. Sakoff, Michela I. Simone, Alister J. Page, Abhi Veerakumarasivam, Edward R. T. Tiekink, Thahira B. S. A. Ravoof

**Affiliations:** 1 Department of Chemistry, Faculty of Science, Universiti Putra Malaysia, 43400 UPM Serdang, Selangor Darul Ehsan, Malaysia; enisnadia89@gmail.com (E.N.M.Y.); aliflatif@upm.edu.my (M.A.M.L.); ibra@upm.edu.my (M.I.M.T.); 2 Discipline of Chemistry, School of Environmental and Life Sciences, University of Newcastle, University Drive, Callaghan, NSW 2308, Australia; michela.simone@newcastle.edu.au; 3 Experimental Therapeutics Group, Department of Medical Oncology, Calvary Mater Newcastle Hospital, Edith Street, Waratah NSW 2298, Australia; jennette.sakoff@newcastle.edu.au; 4 Priority Research Centre for Chemical Biology & Clinical Pharmacology, University of Newcastle, University Drive, Callaghan, NSW 2308, Australia; 5 Department of Biological Sciences, School of Science and Technology, Sunway University, No. 5 Jalan Universiti, 47500 Bandar Sunway, Selangor Darul Ehsan, Malaysia; abhiv@sunway.edu.my; 6 Medical Genetics Laboratory, Faculty of Medicine and Health Sciences, Universiti Putra Malaysia, 43400 UPM Serdang, Selangor Darul Ehsan, Malaysia; 7 Research Centre for Crystalline Materials, School of Science and Technology, Sunway University, No. 5 Jalan Universiti, 47500 Bandar Sunway, Selangor Darul Ehsan, Malaysia; edward.tiekink@gmail.com; 8 Materials Synthesis and Characterization Laboratory, Institute of Advanced Technology, Universiti Putra Malaysia, 43400 UPM Serdang, Selangor Darul Ehsan, Malaysia

**Keywords:** tridentate ONS Schiff bases, organotin(IV), dithiocarbazate, five-coordinate compounds, single-crystal X-ray diffraction analysis, cytotoxic activity, mechanistic studies, molecular docking

## Abstract

Six new organotin(IV) compounds of Schiff bases derived from *S*-R-dithiocarbazate [R = benzyl (B), 2- or 4-methylbenzyl (2M and 4M, respectively)] condensed with 2-hydroxy-3-methoxybenzaldehyde (oVa) were synthesised and characterised by elemental analysis, various spectroscopic techniques including infrared, UV-vis, multinuclear (^1^H, ^13^C, ^119^Sn) NMR and mass spectrometry, and single crystal X-ray diffraction. The organotin(IV) compounds were synthesised from the reaction of Ph_2_SnCl_2_ or Me_2_SnCl_2_ with the Schiff bases (S2MoVaH/S4MoVaH/SBoVaH) to form a total of six new organotin(IV) compounds that had a general formula of [R_2_Sn(L)] (where L = Schiff base; R = Ph or Me). The molecular geometries of Me_2_Sn(S2MoVa), Me_2_Sn(S4MoVa) and Me_2_Sn(SBoVa) were established by X-ray crystallography and verified using density functional theory calculations. Interestingly, each experimental structure contained two independent but chemically similar molecules in the crystallographic asymmetric unit. The coordination geometry for each molecule was defined by thiolate-sulphur, phenoxide-oxygen and imine-nitrogen atoms derived from a dinegative, tridentate dithiocarbazate ligand with the remaining positions occupied by the methyl-carbon atoms of the organo groups. In each case, the resulting five-coordinate C_2_NOS geometry was almost exactly intermediate between ideal trigonal-bipyramidal and square-pyramidal geometries. The cytotoxic activities of the Schiff bases and organotin(IV) compounds were investigated against EJ-28 and RT-112 (bladder), HT29 (colon), U87 and SJ-G2 (glioblastoma), MCF-7 (breast) A2780 (ovarian), H460 (lung), A431 (skin), DU145 (prostate), BE2-C (neuroblastoma) and MIA (pancreatic) cancer cell lines and one normal breast cell line (MCF-10A). Diphenyltin(IV) compounds exhibited greater potency than either the Schiff bases or the respective dimethyltin(IV) compounds. Mechanistic studies on the action of these compounds against bladder cancer cells revealed that they induced the production of reactive oxygen species (ROS). The bladder cancer cells were apoptotic after 24 h post-treatment with the diphenyltin(IV) compounds. The interactions of the organotin(IV) compounds with calf thymus DNA (CT-DNA) were experimentally explored using UV-vis absorption spectroscopy. This study revealed that the organotin(IV) compounds have strong DNA binding affinity, verified via molecular docking simulations, which suggests that these organotin(IV) compounds interact with DNA via groove-binding interactions.

## 1. Introduction

The International Agency for Research on Cancer reported a global incidence of 18.1 million new cancer cases and 9.6 million deaths in 2018, with the number of new cases expected to increase rapidly over the next two decades [1,2]. Chemotherapy remains the most widely-used cancer treatment, as compared to surgery, radiation therapy, immunotherapy, hormone therapy and targeted therapy. Cisplatin is a long-standing effective chemotherapy agent for a number of cancers including testicular, ovarian, cervical, head and neck, bladder, lung and colorectal cancer [3,4]. Platinum-based compounds such as carboplatin, oxaliplatin, nedaplatin and laboplatin are also effective chemotherapeutic agents against tumours [5]. A probable mechanism of action for cisplatin is via the interaction with DNA though the formation of covalent adducts [4]. However, platinum drugs have been known to cause various side-effects including anaemia, diarrhoea, alopecia, petechiae, fatigue, nephrotoxicity, emetogenesis, ototoxicity, neurotoxicity and increasing chemotherapeutic resistance [6]. This has sparked wide-spread research in the synthesis and evaluation of novel non-platinum-based complexes as alternative chemotherapeutic agents.

In recent years, metal compounds with a stable *d*^10^ electronic configuration have received considerable attention due to the wide variety of structures formed that depend on the coordinating ligands and synthetic procedures [7,8,9,10,11,12,13,14,15,16]. Organotin(IV) compounds have found use in industry, medicine and agriculture as stabilisers in polyvinylchloride (PVC) plastics, catalysts [17], anti-fouling paints, fungicides [10], wood preservatives and disinfectants [18]. Organotin(IV) compounds have also been reported as effective biocidal agents against environmental toxicants [19,20]. Their medicinal applications (such as anti-tumour, anti-microbial, anti-nematicidal, anti-insecticidal and anti-inflammatory agents) have been explored [7,21,22,23]. The National Cancer Institute (NCI) has tested over 2000 tin-based pharmacological candidates, the largest number ever tested among the metal-containing drug candidates [24].

Many mechanistic studies on organotin(IV) compounds have indicated that these compounds interact with DNA [25,26], one of the main targets in designing cytotoxic drugs and the biological activity of organotin(IV) compounds is believed to arise from their tendency in binding to or cleaving DNA [27,28]. Wang et al. reported that organotin(IV) compounds containing Schiff bases could interact with CT-DNA through intercalation interactions of cytotoxic drugs and serum albumin (which is involved in the transportation of compounds through the blood stream), where the nature and magnitude of these interactions that could significantly influence the pharmacokinetics have also been investigated [29,30]. Organotin(IV) derivatives bind to both nitrogenous DNA bases and phosphate-oxygen of the DNA sugar backbone [31,32]. Organotin(IV) compounds have been reported to bind to glycoprotein or cellular proteins, hence interacting directly with DNA, causing cell death. Tin(IV) compounds have also been found to induce DNA damage by specifically binding to the phosphate backbone of DNA thus leading changes in DNA conformations [33]. 

The activity of organotin(IV) compounds can be readily modified by the choice of ligand coordinated to the central Sn(IV) ion. Organotin(IV) centres coordinated to hetero-donor atoms, especially oxygen, nitrogen and sulphur, have been investigated with a particular focus on their structure-activity correlations [34,35,36]. Coordination of metals to multidentate Schiff bases have, in most cases, enhanced the biological activity of these compounds [37,38,39]. Tin(IV) compounds of Schiff bases containing pyridine have been reported to be particularly biologically relevant [38,40,41].

In order to expand therapeutic potential of organotin(IV) compounds of dithiocarbazate derivatives, and in light of the biological relevance of previously reported organotin(IV) compounds, we report herein the synthesis of three Schiff bases derived from 2-hydroxy-3-methoxybenzaldehyde dithiocarbazate and six of their organotin(IV) compounds. The X-ray crystal structures of dimethyltin(IV) compounds were determined, and their geometries were compared with theoretical DFT calculations at the Becke, 3-parameter, Lee–Yang–Parr (B3LYP) level of theory. The Schiff bases and organotin(IV) compounds were tested for their cytotoxicity via various assays, which was supported by DNA binding studies.

## 2. Results and Discussion

### 2.1. Synthesis

**Scheme 1:**
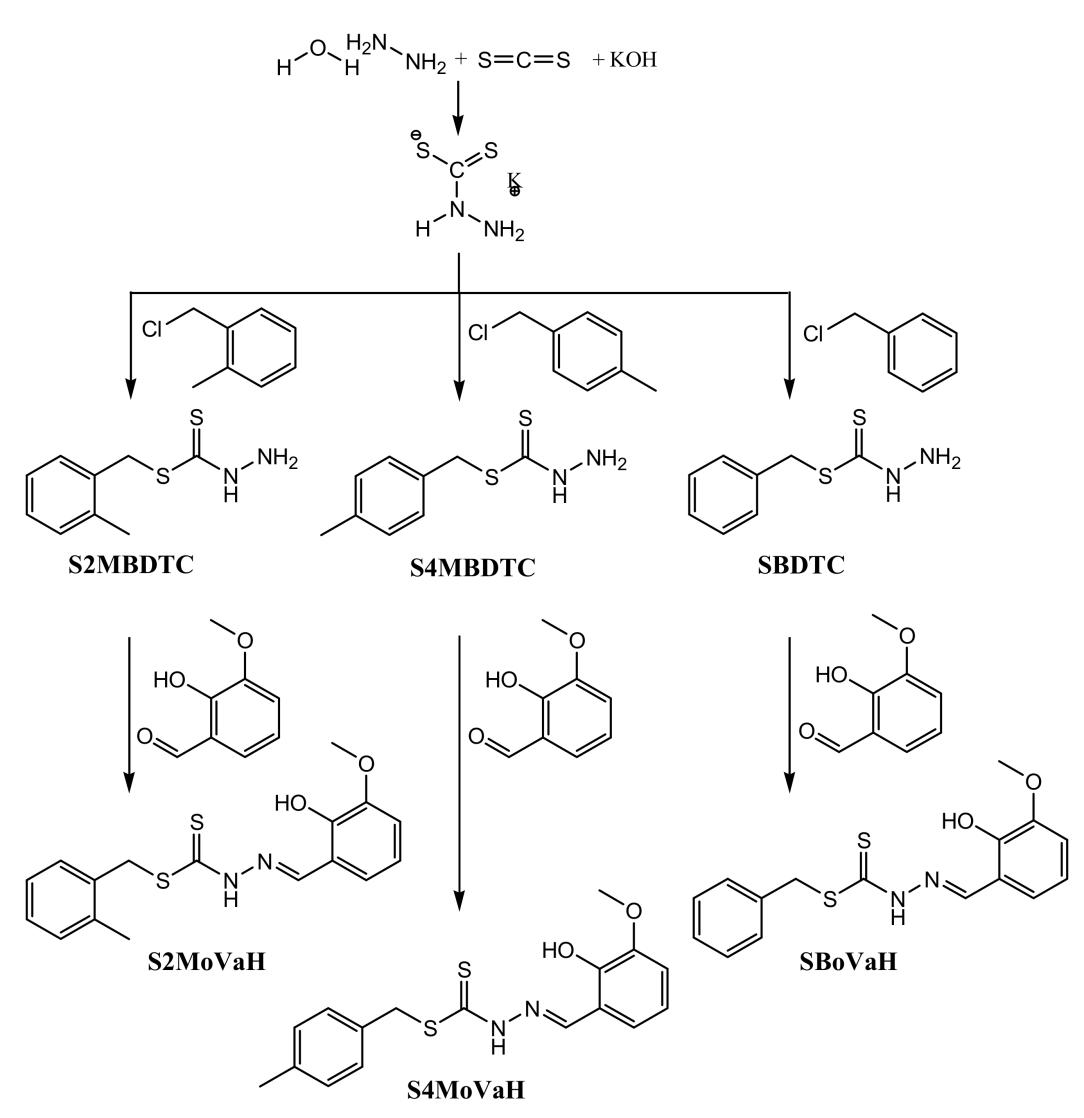
Synthetic pathway for the formation of SBoVaH, S2MoVaH, and S4MoVaH.

**Scheme 2:**
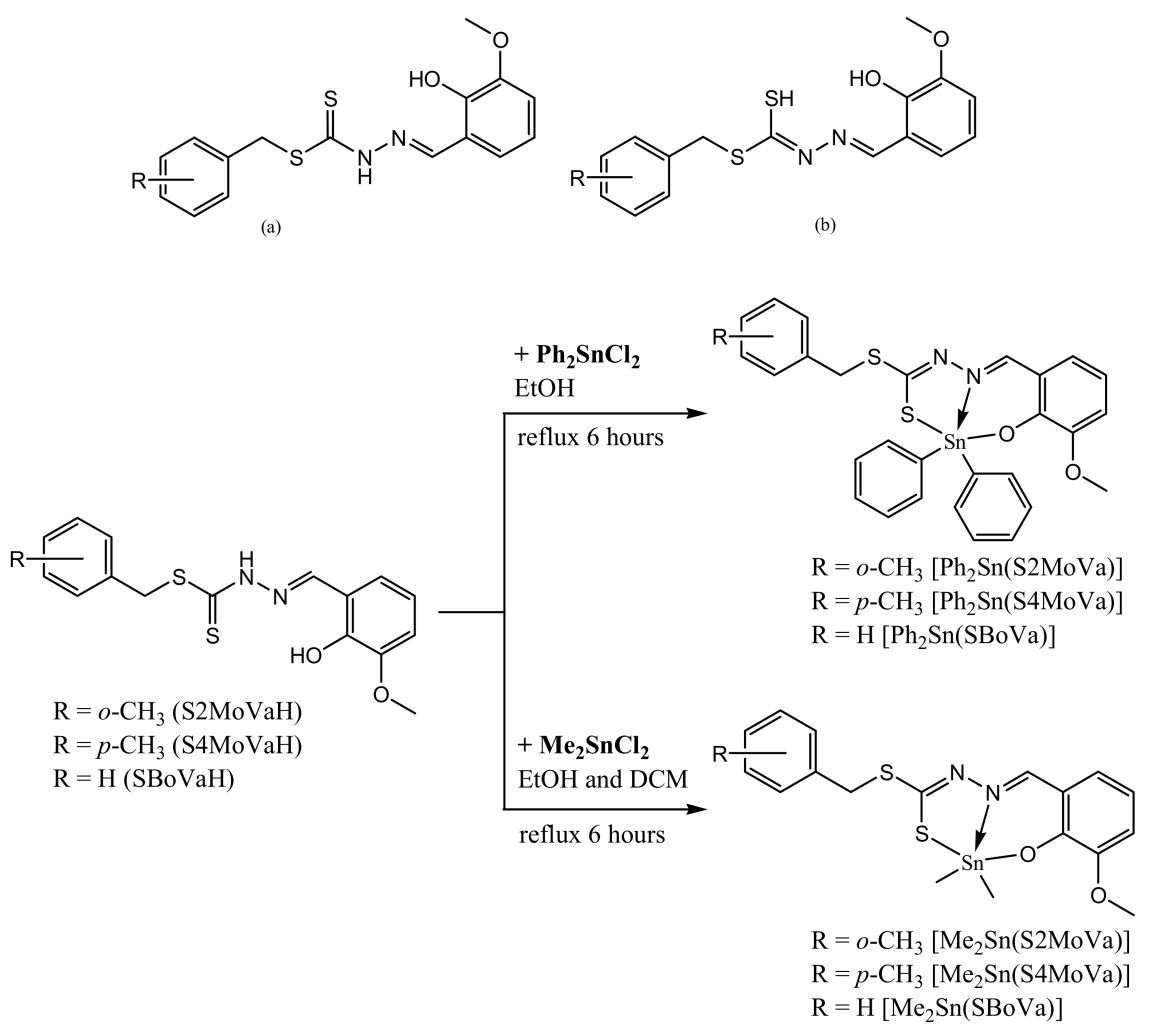
Synthesis of organotin(IV) compounds.

Schiff bases, S2MoVaH, S4MoVaH and SBoVaH, were prepared by reacting *S*-N-R-benzyldithiocarbazates (N = 2,3, R = methyl) and oVa in equimolar ratios (Scheme 1). The Schiff bases were obtained in good yields. The organotin(IV) compounds were synthesised using the corresponding diorganotin(IV) dichloride salts under reflux as shown in Scheme 2. The Schiff bases and their organotin(IV) compounds are yellow, stable at room temperature and soluble in organic solvents (especially chloroform, dimethyl sulfoxide (DMSO) and dimethylformamide (DMF). The room temperature molar conductance values of the organotin(IV) compounds in DMSO at a concentration of 10^−3^ M were in the range of 2.30–8.21 Ω^−1^·cm^2^·mol^−1^, i.e., values well below 25 Ω^−1^·cm^2^·mol^−1^, indicating that these organotin(IV) compounds are non-electrolytes proving the absence of counter-ions, thus indicating that the dithiocarbazate Schiff bases were covalently bonded to the central organotin ion [42].

### 2.2. IR Spectral Analysis

**Figure 1:**
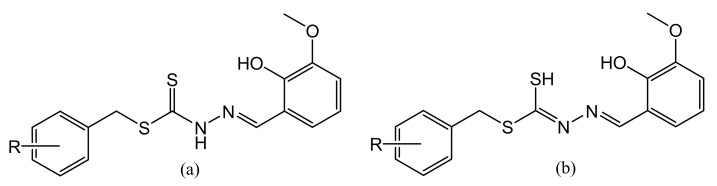
The thione (**a**) and thiol (**b**) tautomeric forms of the Schiff bases (R = *o*–CH_3_, S2MoVaH; *p*-CH_3_, S4MoVaH; R = H, SBoVaH).

The IR spectra indicated the successful formation of the dithiocarbazate Schiff bases and their organotin(IV) compounds. Infrared spectra of Schiff bases and their organotin(IV) compounds were measured in the range 4000 to 280 cm^−1^ and verified using frequencies predicted using density functional theory (DFT). All DFT vibrational frequencies were scaled using a scaling factor of 0.9682 [43] (see Section 3.5.1 for full details of DFT calculations). The Schiff bases have a thioamide(–NH–CS–) functional group and thus, can exist either in the thione (Figure 1a) or thiol (Figure 1b) forms or as a mixture of both thione and thiol tautomeric forms. In the solid-state, the Schiff bases are primarily in the thione form as shown by the presence of the *v*(NH) band at 3089 cm^−1^ and absence of the *v*(SH) band at c.a. 2600 cm^−1^ [44]. Characteristic vibrational frequencies consistent with the successful formation of organotin(IV) compounds have been identified. The *v*(NH) stretching vibration at *ca* 3084–3092 cm^−1^ was absent in the organotin(IV) compounds indicating that the Schiff bases were deprotonated upon complexation. Sharp *v*(C=N) bands corresponding to the Schiff base azomethine group at 1600 (S2MoVaH), 1598 (S4MoVaH) and 1598 (SBoVaH) cm^−1^ were observed to shift to lower wavenumbers upon complexation which indicated coordination via the azomethine nitrogen [45]. The *v*(CSS) splitting bands found in the range 956–964 cm^−1^ provide strong evidence of coordination of the thiolate sulphur atom to the tin ion. The shifting of the hydrazinic *v*(N–N) band to lower wavenumbers in the organotin(IV) compounds showed evidence of coordination via the azomethine nitrogen due to reduction in the repulsion between lone pairs of electrons on the nitrogen atom [46,47,48]. The calculated frequencies correlated well with the experimental frequencies observed in the FT-IR spectra, considering that DFT frequency calculations were performed in the gas-phase. The N–H band in the experimental FTIR spectra of Schiff bases red-shifted owing to intermolecular hydrogen bonding [49]. The absence of hydroxyl stretching in the experimental spectra of the Schiff bases could be due to intermolecular and intramolecular hydrogen bonding as was observed in similar reported organotin(IV) compounds [50]. Moreover, only slight differences were observed in the experimental and calculated data for *v*(C=N), *v*(N–N) and *v*(C=S). Complete IR data are provided in Table S1. 

### 2.3. NMR Spectroscopic Analysis

NMR spectra of the dithiocarbazate Schiff bases were recorded in DMSO-*d*_6_, while the NMR spectra of their organotin(IV) compounds were recorded in CDCl_3_. The data obtained were in good agreement with the expected structures in both solvents. The absence of the exchangeable SH signal in the ^1^H NMR spectra of the Schiff bases indicated the predominance of the thione tautomer in DMSO-*d*_6_ [51]. The spectra of the Schiff bases also exhibited a downfield signal at ~13.32–13.34 ppm due to the NH group. For all organotin(IV) compounds, the ^1^H NMR signal for the azomethine NH proton was absent due to the coordination to the metal ion by the Schiff base, in corroboration with the absence of the *v*(NH) FTIR band noted in Section 2.2. A singlet appeared in the downfield region of the ^1^H NMR spectra of the Schiff bases which corresponded to an *sp*^2^-C–OH proton. Proton chemical shifts for both NH and OH disappeared in the spectra of the organotin(IV) compounds suggesting double deprotonation of the Schiff bases and supporting coordination of both the phenolic-oxygen and -nitrogen atoms of the Schiff base to the tin ion.

The ^13^C NMR spectra of the dithiocarbazate Schiff bases showed a downfield chemical shift at ~196 ppm, indicating the existence of the C=S group and thus the predominance of the thione tautomer in solution. This signal was shifted upfield in the organotin(IV) compounds (170–180 ppm), indicating a decrease in electron density at the C=S carbon atom when the sulphur atom was complexed to the tin ion, consistent with the structures proposed for the organotin(IV) compounds [52]. 

The ^119^Sn NMR spectra of the organotin(IV) compounds were recorded at room temperature in CDCl_3_ using SnMe_4_ as the external standard. Theoretically, as the coordination number about tin increases, chemical shifts in ^119^Sn NMR move towards lower frequencies [53]. ^119^Sn NMR spectra are also very sensitive to the type of donor atom coordinated to the tin and its environment, e.g., alkyl or aryl groups in the case of a C donor and to the bond angles around the tin centre [54]. However, shielding or deshielding of the tin nucleus does not have a significant effect on the chemical shifts [55]. Dimethyltin(IV) compounds showed less negative ^119^Sn chemical shifts compared to diphenyltin(IV) compounds. The chemical shift values obtained were within the range reported for other diphenyl- or dimethyl(IV) compounds with heterocyclic ligands having penta-coordinated trigonal bipyramidal geometry [53,56]. Complete ^119^Sn NMR data are provided in Section 3.3. The NMR details for the Schiff bases and organotin(IV) compounds are tabulated in Tables S2 and S3.

### 2.4. Mass Spectral Analysis

Mass spectral data for the Schiff bases were recorded in DMSO and were found to be consistent with the proposed formulation of the Schiff bases. Mass spectra displayed prominent peaks at *m*/*z* 346.15 and 346.10 for S2MoVaH and S4MoVaH, respectively, corresponding to [C_17_H_18_N_2_O_2_S_2_]^+^, and *m*/*z* 332.10 for SBoVaH corresponding to [C_16_H_16_N_2_O_2_S_2_]^+^.

### 2.5. X-ray Crystallography

**Figure 2:**
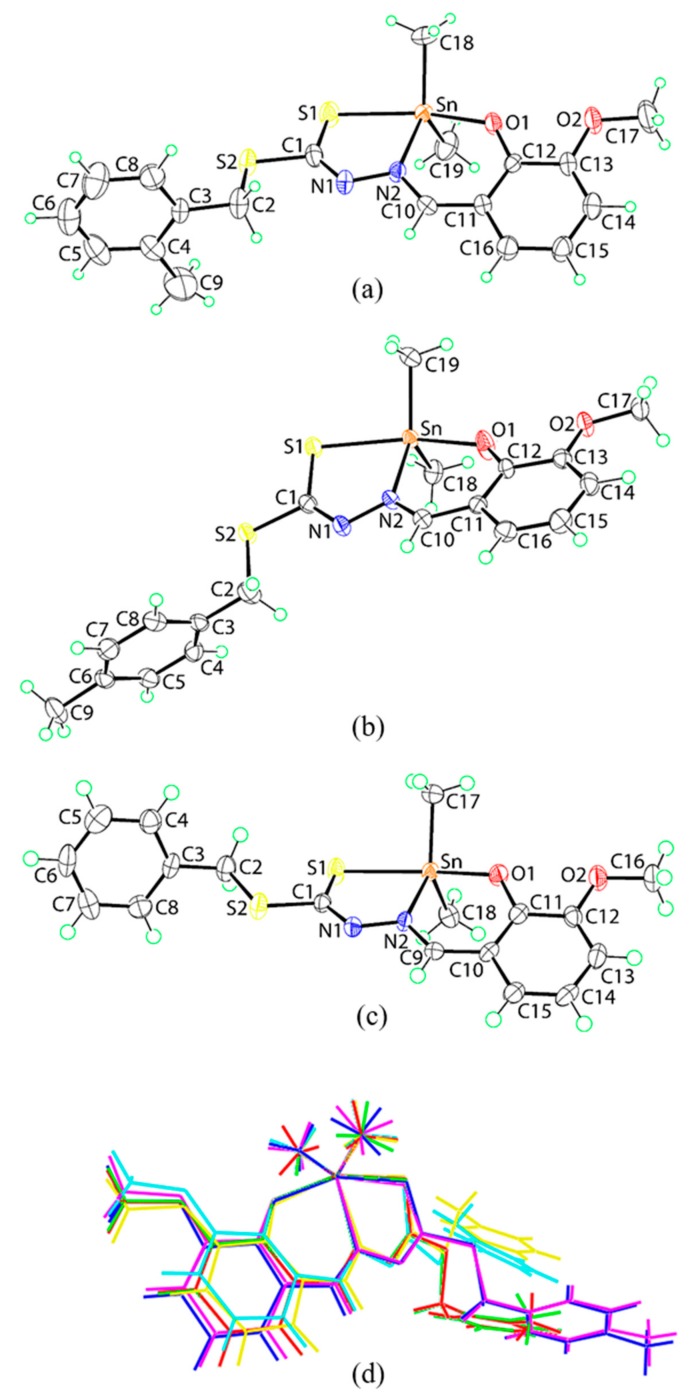
Molecular structures of the first independent molecules of (**a**) Me_2_Sn(S2MoVa), (**b**) Me_2_Sn(S4MoVa) and (**c**) Me_2_Sn(SBoVa) showing atom labelling schemes. (**d**) Overlay diagram of the two independent molecules of each of Me_2_Sn(S2MoVa), a (red image) and b (green), Me_2_Sn(S4MoVa), a (blue) and b (pink), and Me_2_Sn(SBoVa) a (yellow) and b (aqua) with the SnMe_2_ planes superimposed.

**Figure 3:**
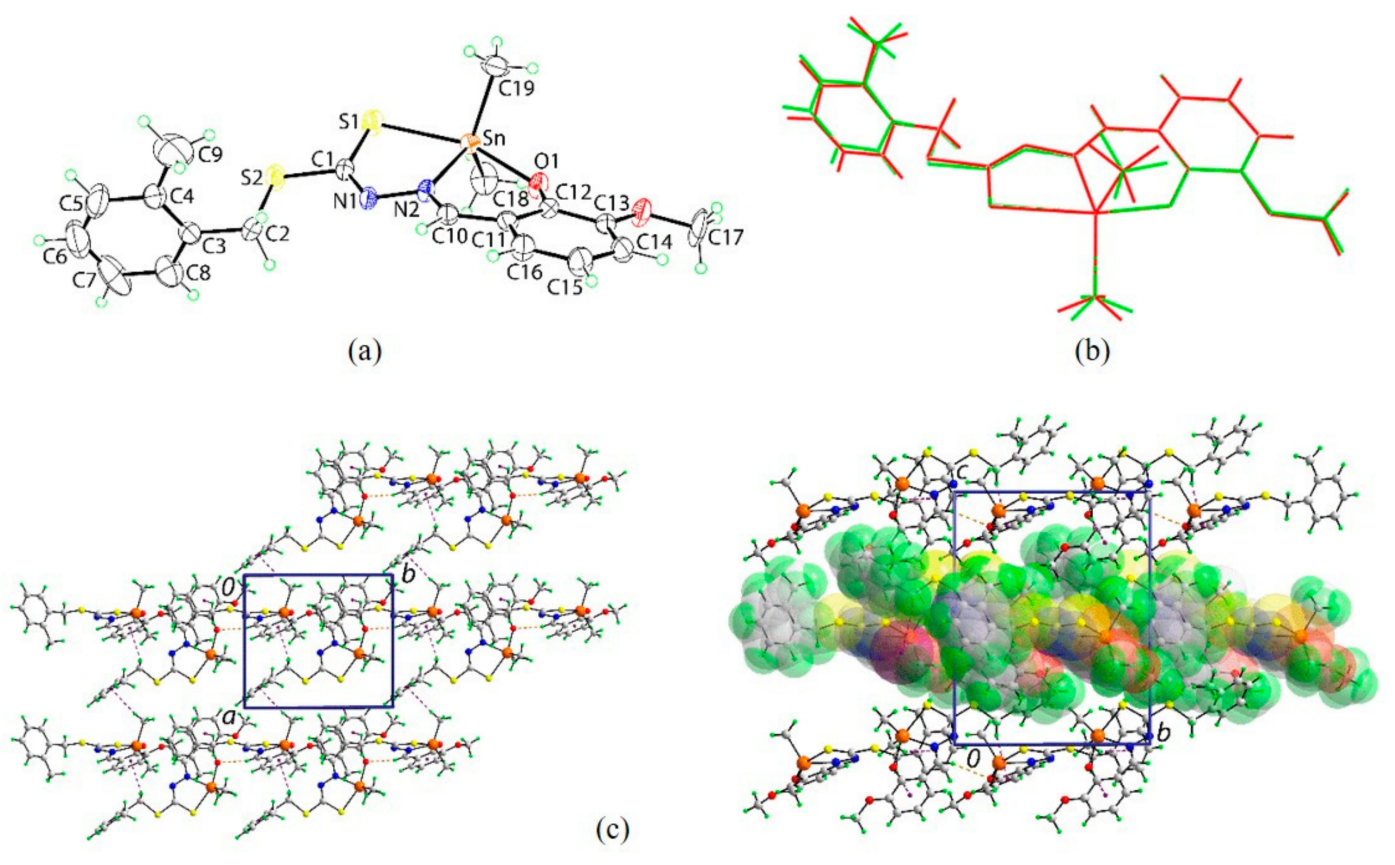
Crystallographic diagrams for Me_2_Sn(S2MoVa): (**a**) Molecular structure of the second independent molecule, molecule b, (**b**) overlay diagram of molecules a (red image) and inverted-b (green) drawn so the SnC_2_ atoms of the Me_2_Sn moiety are overlapped and (**c**) a view of the supramolecular layer in the *ab*-plane (left image) and a view of the unit cell contents in projection down the a-axis with one layer highlighted in space-filling mode (right image). The C–H^…^O and C–H^…^π interactions are shown as orange and purple dashed lines, respectively.

**Figure 4:**
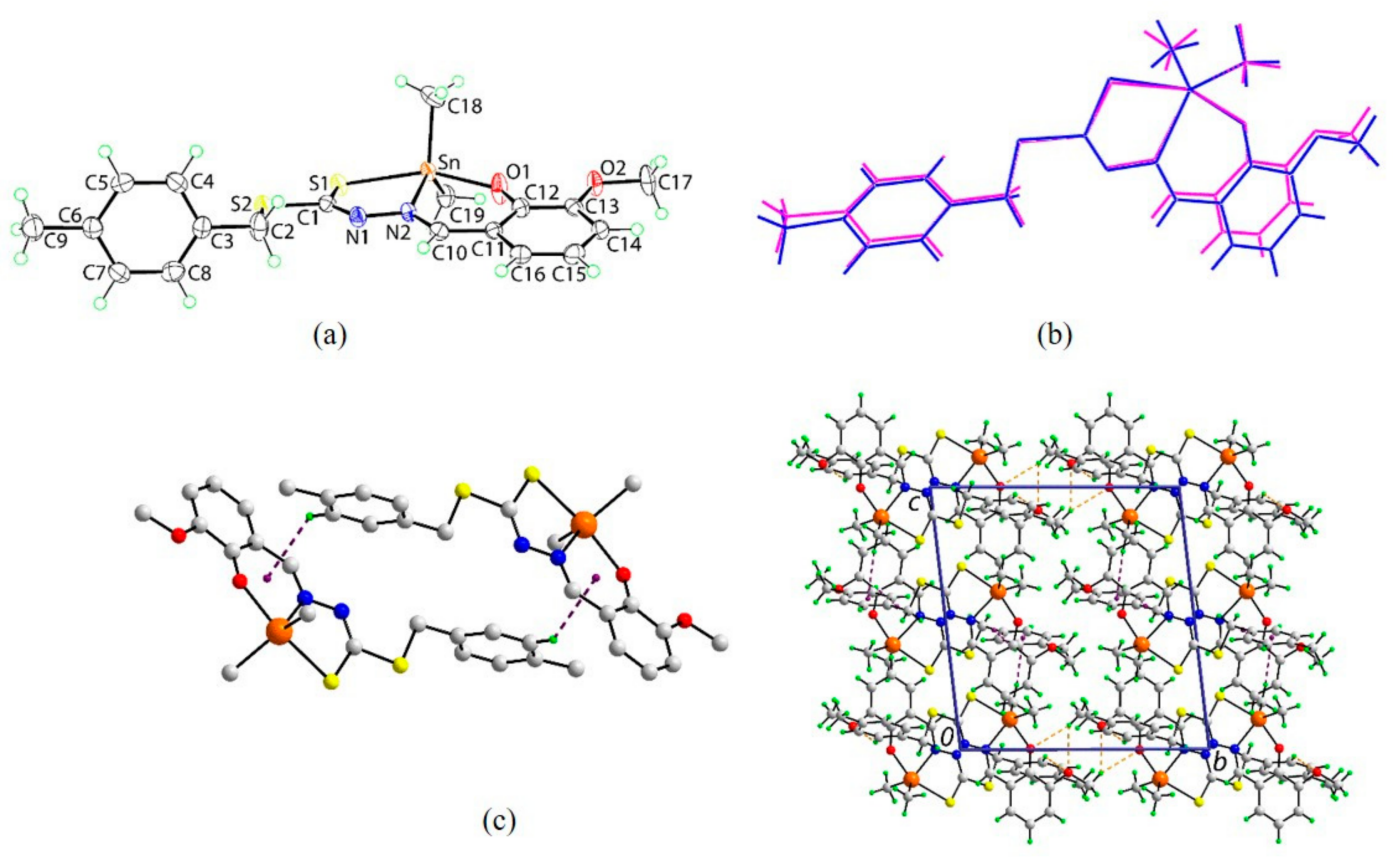
Crystallographic diagrams for Me_2_Sn(S4MoVa): (**a**) Molecular structure of the second independent molecule, molecule b, (**b**) overlay diagram of molecules a (blue image) and inverted-b (pink) drawn so the SnC_2_ atoms of the Me_2_Sn moiety are overlapped and (**c**) a view of the supramolecular dimer sustained by C–H^…^π (chelate) interactions (left image; non-participating hydrogen atoms have been omitted) and a view of the unit cell contents in projection down the a-axis. The C–H^…^O and C–H^…^π interactions are shown as orange and purple dashed lines, respectively.

**Figure 5:**
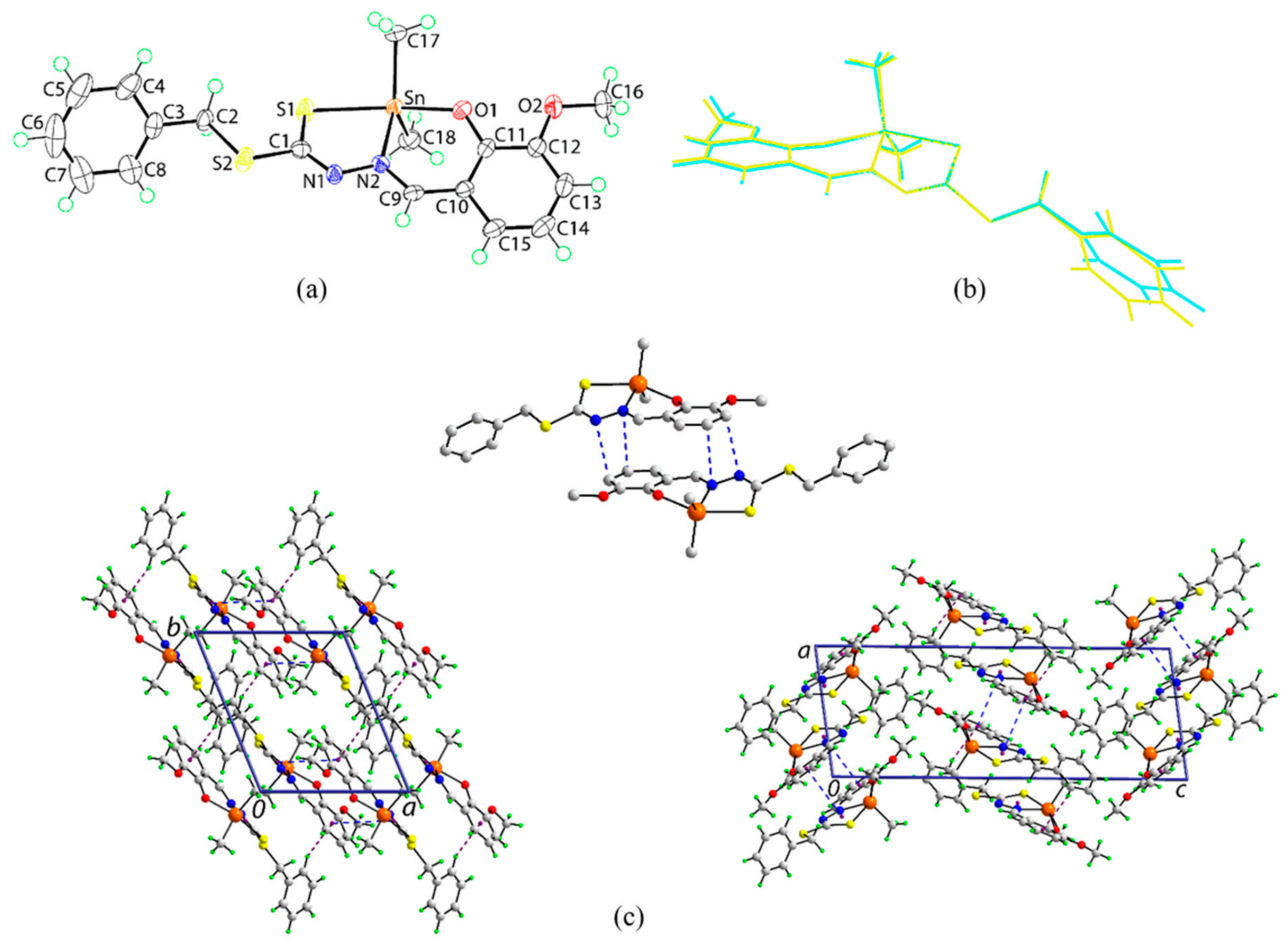
Crystallographic diagrams for Sn(SBoVa)Me_2_: (**a**) Molecular structure of the second independent molecule, molecule b, (**b**) overlay diagram of molecules a (yellow image) and b (aqua) drawn so the five-membered rings are overlapped and (**c**) supramolecular dimer sustained by edge-to-edge chelate ring^…^benzene interactions (upper image) between Sn1-containing molecules, supramolecular layer sustained by edge-to-edge π (chelate ring)^…^π (ethoxybenzene) and phenyl-C–H^…^π (methoxybenzene) interactions occurring between Sn2-containing molecules and a view of the unit cell contents in projection down the b-axis. The edge-to-edge π (chelate ring)^…^π (ethoxybenzene) and C–H^…^π interactions are shown as blue and purple dashed lines, respectively.

The crystal structures of Me_2_Sn(S2MoVa), Me_2_Sn(S4MoVa) and Me_2_Sn(SBoVa), each featured two independent molecules in the crystallographic asymmetric unit, i.e., molecules a and b. Figure 2a–c shows the molecular structures for the first independent molecule in each crystal and images for the second independent molecules are shown in Figure 3a, Figure 4a and Figure 5a; selected geometric parameters are collated in Table 1. The major conformational difference between the molecules in each of Me_2_Sn(S2MoVa), Me_2_Sn(S4MoVa) and Me_2_Sn(SBoVa) is highlighted in the overlay diagram shown in Figure 2d. Whereas in the tolyl species, the tolyl substituent is orientated to be directed away from the coordinated S1 atom, i.e., can be considered anti to S1, in each molecule of Me_2_Sn(S4MoVa), the phenyl ring is syn to the S1 atom.

The tin atom in Me_2_Sn(S2MoVa) is coordinated by the thiolate-S1, phenoxide-O1 and imine-N atoms derived from the dinegative, [(3-methoxy-2-oxyphenyl)methyl] dithiocarbazate ligand. The five-coordinate geometry is completed by a carbon atom from each of the two methyl groups. The assignment of thiolate-S1 is readily made by comparing the length of the C1‒S1 bond in Me_2_Sn(S2MoVa), i.e., 1.726(9) and 1.717(8) Å, for the two independent molecules comprising the asymmetric unit, with that in the uncoordinated Schiff base (which lacks the 2-methyl group in the ester group) of 1.670(2) Å [50], i.e., the bond lengths in Me_2_Sn(S2MoVa) are significantly longer. The C1‒S2 bond lengths are experimentally equivalent at 1.744(8) and 1.747(8) Å [50]. However, the two C1=N1 imine bond lengths in Me_2_Sn(S2MoVa) are 1.304(11) and 1.273(10) Å, which are significantly longer than the equivalent C1–N1(H) bond of 1.338(2) Å in the free Schiff base [50]. The mode of coordination of the dithiocarbazate ligand leads to the formation of five- and six-membered chelate rings, with the chelate angle in the former being approximately 5° more acute than that in the latter, Table 1. The Sn, S1, N1, N2, C1 chelate ring in molecule a is non-planar and is twisted about the Sn‒S1 bond. On the other hand, the Sn, O1, N1, C10–C12 chelate ring is best described as having an envelope conformation whereby the Sn atom lies 0.528(11) Å above the plane. However, the structure of the second independent molecule in the asymmetric unit, molecule b, is slightly different whereby the five-membered ring is an envelope with the Sn atom lying 0.372(12) Å above the plane defined by the remaining four atoms in this ring [r.m.s. deviation = 0.008 Å]. The six-membered ring is also an envelope with the Sn atom lying 0.528(11) Å out of the plane defined by the five remaining atoms which present a r.m.s. deviation = 0.022 Å. The dihedral angle between the best planes through the chelate rings in the molecule a is 11.12(17)° while for molecule b, the equivalent angle is 12.4(3)°. The benzyl ring is almost orthogonal to the five-membered chelate ring for both independent molecules in the unit cell, forming dihedral angles of 88.3(3) and 86.8(3)°, respectively. Similarly, the dihedral angles between the outer rings are 72.3(3) and 75.5(3)°, respectively, also indicating an approximate orthogonal relationship. The similarity between the conformations found for the two independent molecules in the asymmetric unit is highlighted in the overlay diagram shown in Figure 3. The five-coordinate geometry defined by the C_2_NOS donor set for molecule a, with five-coordinate geometry descriptor τ = 0.48, is intermediate between ideal square-pyramidal (τ = 0.0) and trigonal-bipyramidal geometries (τ = 1.0) [57]; the value of τ is 0.50 for molecule b. For each independent molecule, the widest angle corresponds to the S1‒Sn‒O1 angle with the second widest angle being subtended by the tin-bound methyl groups. The angles about the quaternary-C1 atom span nearly 20° with the widest angles involving the doubly bonded N1 atom. Of these, the angle involving the S1 atom, being the widest, is consistent with some double bond character in the C1‒S1 bond. Consistent with this, (see Table 1), the C1‒S1 bond lengths are systematically shorter than the C1‒S2 bonds.

The isomeric compound, Me_2_Sn(S4MoVa), having an ester 4-tolyl residue rather than 2-tolyl, presents very similar characteristics to those exhibited in the structure of Me_2_Sn(S2MoVa) (Figure 2b and Figure 4, Table 1). The conformations of the two chelate rings in the independent molecules are the approximately same, being based on an envelope conformation with the tin atom being the flap in all cases. The envelope is more pronounced, cf. Me_2_Sn(S2MOVa), in the five-membered rings with the tin atom lying 0.486(4) Å [0.490(4) Å for molecule b] above the plane of the four remaining atoms which have a r.m.s. deviation = 0.009 Å [0.012 Å] but, is somewhat flattened in the six-membered rings with the tin atom lying 0.187(4) Å [0.132(4) Å] out of the plane defined by the five remaining atoms with a r.m.s. deviation = 0.013 Å [0.017 Å]. The dihedral angle between the best planes through the chelate rings is 10.24(8)° [8.53(11)°]. Near orthogonal relationships between the five-membered chelate rings and the pendant tolyl rings persist, and the dihedral angles between the outer phenyl rings is 65.75(10)° [78.30(10)°]. The value of τ is 0.54 [0.41]. A difference is noted in the coordination geometries in that the second widest angles at the tin atom, after S1–Sn–O1, are subtended by the N2 and C19 atoms, i.e., 125.83(12)° [131.09(12)°], rather than the C18–Sn–C19 angle, Table 1.

The unsubstituted analogue of the aforementioned isomeric structures, i.e., Me_2_Sn(SBOVa), is similar to those just described (Figure 2c and Figure 5, Table 1). A likely trend in the Sn‒S1 bond lengths may be discerned, Table 1, in that these are longest in Me_2_Sn(SBOVa) compared to the tolyl derivatives, Me_2_Sn(S2MOVa) and Me_2_Sn(S4MOVa), pointing to an electronic influence, i.e., activation of the aromatic ring owing to the presence of electron-donating methyl groups; while there is evidence for shorter Sn‒S1 bond lengths in Me_2_Sn(S4MOVa) cf. Me_2_Sn(S2MOVa), the standard uncertainty values in the latter preclude a definitive statement on this although the trend for shorter Sn‒S1 bonds in Me_2_Sn(S4MOVa) is consistent with expectation. The conformational flexibility in the chelate ligands formed by these tridentate ligands is reflected in the flattened envelope conformations for the five-membered rings with the Sn atom lying 0.326(6) Å above the plane defined by the four remaining atoms [r.m.s. deviation = 0.021 Å; comparable parameters for molecule b are 0.287(6) and 0.024 Å, respectively]. By contrast, pronounced envelope conformations are found for the six-membered rings [0.675(5) and 0.043 Å for molecule a; 0.706(5) and 0.048 Å for molecule b]. The dihedral angle between the best planes through the chelate rings is 13.66(16)° [13.92(17)°]. The values of τ for the independent molecules compute to 0.47 and 0.44, respectively. As for Me_2_Sn(S4MOVa), after the S1–Sn–O1 angles, the widest angle subtended at the tin atom in Me_2_Sn(SBOVa) for the second independent molecule involves the coordinated imine-N2 and a tin-bound methyl-C17 atoms, i.e., 127.66(17)°. Of the three new structures reported herein, the greatest agreement between the two independent molecules comprising the asymmetric unit is found for Me_2_Sn(S4MOVa), see Figure 5 and Table 1.

In the absence of conventional hydrogen bonding interactions, the crystals of Me_2_Sn(S2MoVa), Me_2_Sn(S4MoVa) and Me_2_Sn(SBoVa) feature a range of weak non-covalent interactions. Packing diagrams are given in Figure 3c, Figure 4c and Figure 5c and geometric details characterising the specified intermolecular interactions are given in Tables S4–S6. In the molecular packing of Me_2_Sn(S2MoVa), imine-C–H^…^O(phenoxide), methylene-C–H^…^π(methoxybenzene), involving both methylene groups, and tin-bound-methyl-C–H^…^π (tolyl) interactions combine to stabilise supramolecular layers in the *ab*-plane; layers stack without directional interactions between them. The crystal of Me_2_Sn(S4MoVa) features additional recognisable points of contact between molecules largely due to the participation of the methoxy substituents. Thus, tolyl-C–H^…^O (methoxy) and methoxy-methyl-C–H^…^O (methoxy, phenoxide) interactions are apparent as are tolyl-C5–H^…^π(chelate) and tin-bound-methyl-C–H^…^π(tolyl) points of contact. These consolidate the three-dimensional architecture, see Figure 4c. The presence of C–H^…^π (chelate) interactions is of interest and have only relatively recently been appreciated as being important in contributing to the stability of coordination compounds [58,59,60]. These interactions usually stabilise (centrosymmetric) dimeric aggregates [59] and that is the case in Me_2_Sn(S4MoVa), see Figure 4c. In the crystal of Me_2_Sn(SBoVa), both phenyl-C–H^…^π (methoxybenzene) and less common π(chelate)^…^π(methoxybenzene) interactions are formed. The latter interactions have been the subject of a recent comprehensive review [61]. In the present case, Sn1-containing molecules interact in this mode via association between the five-membered (Sn/S1/N1/N2/C1) chelate ring and a methoxybenzene ring. However, these interactions are best described as edge-to-edge interactions with the closest contact occurring between a nitrogen atom of the chelate ring and the benzene-carbon, see Figure 5c. Similar edge-to-edge contacts are found for the second independent molecule with the resulting dimeric aggregates being connected into a supramolecular layer in the *ab*-plane by phenyl-C–H^…^π (methoxybenzene) interactions. Globally, the packing comprises alternating layers of molecules of a (with no obvious points of contact between the dimeric units) and molecules of b along the *c*-axis with no directional interactions between successive layers.

B3LYP-derived bond lengths and angles of Me_2_Sn(S2MoVa), Me_2_Sn(S4MoVa) and Me_2_Sn(SBoVa) are in excellent agreement with the values obtained from the crystallographic analysis above (see Table 1), consistent with the absence of strong directional intermolecular interactions in their crystals. For instance, the deviations in the selected bond lengths/angles of Me_2_Sn(S2MoVa), Me_2_Sn(S4MoVa) and Me_2_Sn(SBoVa) ranged from 0.002–0.144 Å/0–5.6°, whereby the maximum bond length deviation corresponded to the Sn–N2 bond for Me_2_Sn(S4MoVa) and the maximum bond angle deviation corresponded to the S1–Sn–O1 angle for Me_2_Sn(SBoVa).

### 2.6. UV-Vis Absorption Spectroscopy

The experimental UV-vis spectra of the Schiff bases measured in DMSO exhibited prominent absorption bands at 340–348 and 371–389 nm. The corresponding peaks were predicted at 334 and 377–378 nm using B3LYP. The highest occupied molecular orbital and lowest unoccupied molecular orbital (HOMO-LUMO) electron density distribution of the Schiff bases and organotin(IV) compounds (see Figure S1) showed that the HOMO was predominantly centred on the thiolate group and azomethine moieties of the dithiocarbazate backbone in each of the compounds investigated here. Conversely, the LUMO was consistently centred on the 2-hydroxy-3-methoxyphenyl ring, as well as on the backbone of the dithiocarbazate moieties. Thus, the 334 nm absorption peak was attributed to the n→π* transitions associated with the non-bonding electron pair of the azomethine nitrogen and sulphur atoms. There was some π→π* character to this transition as well, relating to electron delocalization in the aromatic rings. In the spectra of the organotin(IV) compounds, the additional, strong S→Sn^IV^ intra-ligand band indicated coordination at 444–447 nm. The presence of the S→Sn^IV^ LMCT band was corroborated by IR evidence which showed that the tin centre was coordinated to the sulphur atom in the organotin(IV) compounds [62]. The HOMO and LUMO of the organotin(IV) compounds were comparable to those of the corresponding Schiff bases, i.e., the HOMO was largely centred on the backbone of the Schiff bases and the LUMO mostly covered the 2-hydroxy-3-methoxyphenyl ring as well as the backbone of the Schiff base thus explaining the observed n→π* and π→π* transitions at 307–315 and 353–373 nm, respectively. The HOMO and LUMO transitions were the same for all the Schiff bases due to their similar structures, the difference being only the presence and position of the methyl group attached to the phenyl ring. Complete experimental and calculated UV-vis data are listed in Table S7. 

### 2.7. Biological Assays

#### 2.7.1. Cytotoxicity

**Figure 6:**
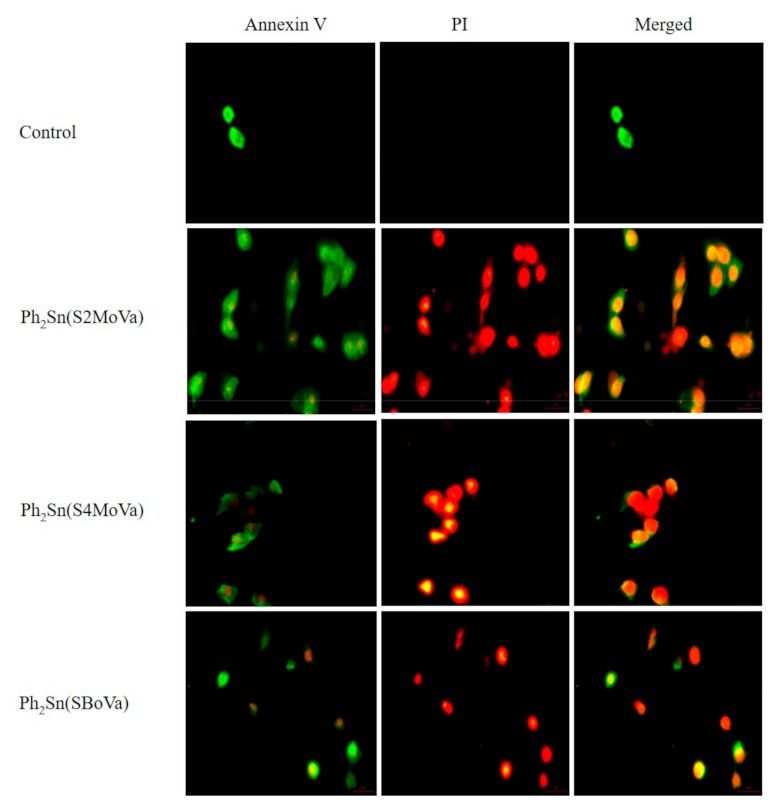
Apoptosis detection through fluorescence microscopy. Cells were treated for 24 h with Ph_2_Sn(SBoVa) (0.58 µM), Ph_2_Sn(S2MoVa) (0.31 µM) and Ph_2_Sn(S4MoVa) (1.66 µM) and the negative control (DMSO) in complete media. After staining with Annexin V and PI, necrotic and apoptotic cells were detected by fluorescence microscopy (20×).

The Schiff bases and their organotin(IV) compounds were screened for in vitro cytotoxicity against a panel of 12 cancer cell lines: EJ-28 (muscle invasive bladder), RT-112 (minimum-invasive bladder), HT29 (colon), U87 and SJ-G2 (glioblastoma), MCF-7 (breast), A2780 (ovarian), H460 (lung), A431 (skin), DU145 (prostate), BE2-C (neuroblastoma), MIA (pancreatic) and one normal breast cell line (MCF-10A) using the MTT metabolic assay (Figure 6). After 72 h of incubation, a dose-dependent proliferative effect towards the cancer cell lines was measured at very low micromolar (µM) concentrations of the compounds. The growth inhibition concentration of the Schiff bases and their organotin(IV) compounds required to inhibit 50% cell proliferation relative to the cells that were treated with DMSO (control) are given in Table 2. The stability of these compounds was assessed in DMSO as well as in a mixture of DMSO-H_2_O. The absorbance obtained from UV-vis spectroscopy analysis was monitored for 72 h, and an unchanged pattern in the spectra was indicative that the compounds were stable in both the solvent systems tested. 

As outlined earlier, the effect of the substitution in the methyl group on the dithiocarbazate backbone on the coordination with the tin ion was explored. The GI_50_ values for the three Schiff bases showed a slight difference; the presence of methyl group at *ortho* (S2MoVaH) and *para* (S4MoVaH) positions resulted in a slight reduction in potency as compared to SBoVaH. This cytotoxicity difference was probably due to the hydrophobic nature of the methyl group [63]. To note, S4MoVaH exhibited the lowest potency against HT29 and MIA with GI_50_ values of 8.0 ± 3.5 and 11 ± 2.3 µM, respectively. It was also observed that the coordination of the Schiff bases with diphenyltin(IV) induced potent selective growth inhibition in A2780, BE2-C and MIA cells, in which the compounds exhibited potency higher than their respective Schiff bases. Cytotoxic selectivity was also observed for Ph_2_Sn(SBoVa) and Ph_2_Sn(S4MoVa) against MCF-7 with GI_50_ values of 0.90 ± 0.16 and 0.82 ± 0.14 µM, respectively. This constituted a potency that was almost thrice higher than that of the corresponding Schiff bases, SBoVaH and S4MoVaH which had values of 2.5 ± 0.12 and 3.5 ± 0.00 µM, respectively. In addition, Ph_2_Sn(S4MoVa) exhibited a much higher potency (GI_50_ value of 0.87 ± 0.22 µM) than S4MoVaH (GI_50_ value of 8.0 ± 3.5 µM) against HT-29 cancer cells. A 3-fold greater activity against SJ-G2 was observed for Ph_2_Sn(SBoVa) and Ph_2_Sn(S2MoVa) with GI_50_ values of 0.74 ± 0.15 and 0.84 ± 0.09 µM respectively, than their respective Schiff bases. It is noteworthy to mention that the diphenyltin(IV) compounds showed high selectivity against EJ-28, RT-112, MCF-7, A2780, BE2-C, SJ-G2 and MIA cell lines in the range 1.4-5.0-fold as compared to the normal breast cell line (MCF10A). 

By contrast, dimethyltin(IV) compounds showed almost similar cytotoxicity values when assayed against the panel of cancer cell lines suggesting that the presence of two phenyl groups coordinated directly to the tin ion improved the potency of the Schiff bases. Electron delocalisation over the chelate rings, which increased the lipophilicity of the molecules and favoured their permeation through the lipid layer of the cancer cell membranes could also be a contributing factor to the enhancement of cytotoxicity [64]. A third contributing factor could be the ease of ability of the phenyl planes to intercalate into the base pairs of DNA in the cancer cell lines [8]. To better understand the mode of biological action of the compounds that showed promising activity, apoptosis assays using the minimally invasive bladder cancer RT-112 cells and DNA binding studies were carried out by evaluating the compounds’ mechanism of action.

#### 2.7.2. Annexin V Assays of Ph_2_Sn(S2MoVa)Ph_2_, Ph_2_Sn(S4MoVa), and Ph_2_Sn(SBoVa)-Treated RT-112 Cells

Cell death usually occurs by apoptosis or necrosis, the latter induces severe inflammation. The apoptotic cells containing apoptotic bodies (small membrane-bound vesicles), are engulfed by macrophages, and hence no inflammatory response is triggered. On this account, as apoptotic cell induction is considered crucial in cytotoxic drug development, investigations using the Annexin V assay were performed to study the apoptosis effect on cancer cells of the organotin(IV) compounds. The three most highly cytotoxic diphenyltin(IV) compounds at their specific IC_50_ concentrations were chosen for this assay. The analysis was carried out using RT-112, minimum-invasive bladder cancer cells with high levels of epidermal growth factor receptor expression. In the early stages of apoptosis, changes occurred at the cell surface [65]. The plasma membrane alteration occurs due to the translocation of phosphatidyl-serine (PS) from the inner part of the plasma membrane where it is normally located on the cytoplasmic surface, to the outer layer. The PS exposed on the cell surface has high binding affinity to the Annexin V. In the later stages of apoptosis, as well as necrosis, the cell membranes lose their membrane integrity and allows propidium iodide (PI) to enter into the nucleus of the cells. Annexin V/PI double staining was used to differentiate viable cells (Annexin V^−^ and PI^−^) in early apoptosis (Annexin V^+^ and PI^−^) or late apoptosis (Annexin V^+^ and PI^+^) and necrosis (annexin V^−^ and PI^+^) stages [66]. Under fluorescence microscopy, the cells stained by annexin V appear green, whereas the cells stained by PI appear red. After 24 h exposure to the diphenyltin(IV) compounds, Figure 6 show that the cells undergoing late apoptosis and necrosis features. Thus, this assay proved that the diphenyltin(IV) compounds trigger the activation of apoptosis [67].

#### 2.7.3. Measurement of Reactive Oxygen Species (ROS) in RT-112 Cells Treated with Ph_2_Sn(S2MoVa) and Ph_2_Sn(S4MoVa)

**Figure 7:**
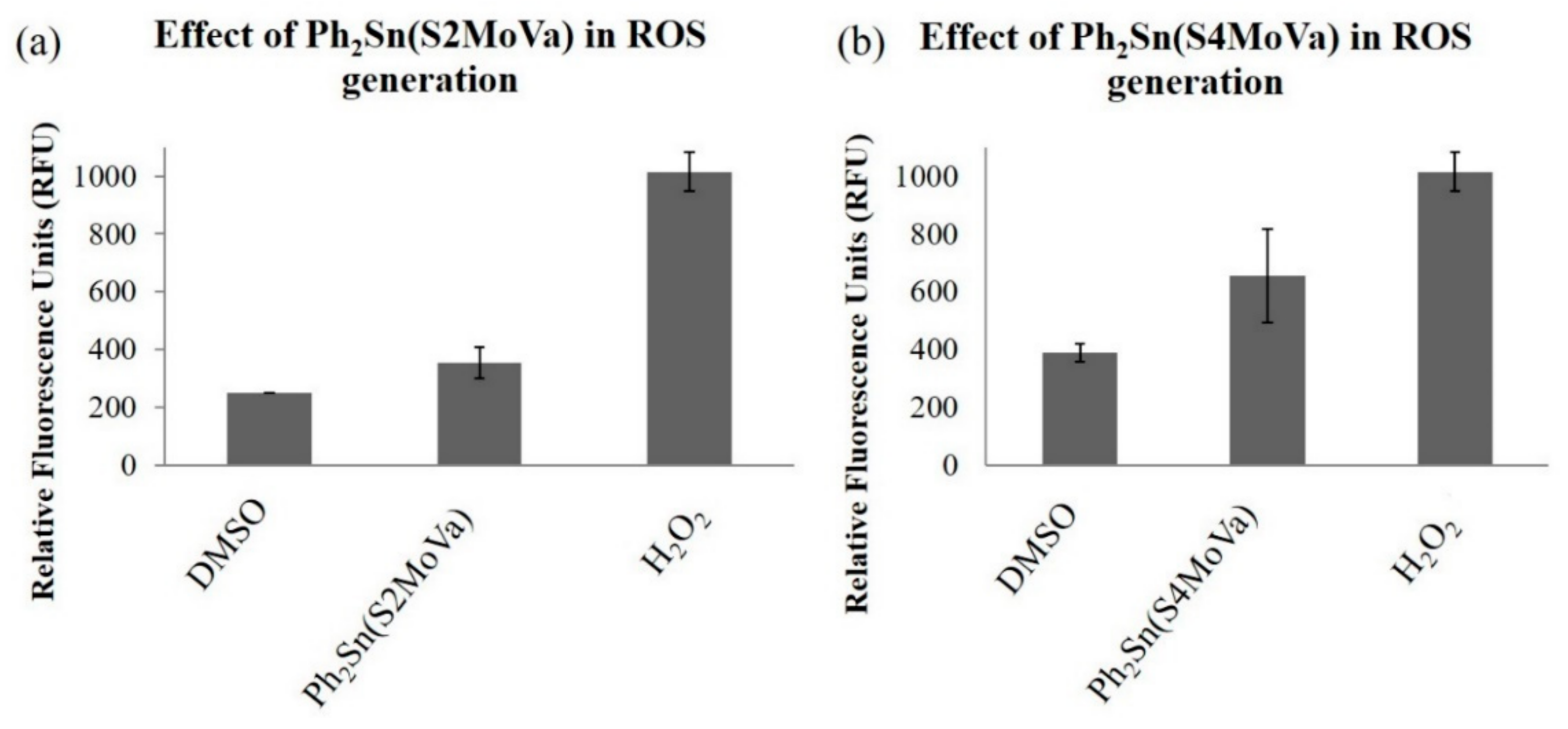
Percent reactive oxygen species (ROS) production in RT-112 cells treated with (**a**) Ph_2_Sn(S2MoVa) (0.31 μM) (**b**) Ph_2_Sn(S4MoVa) (1.66 μM) for 24 h and stained with 1 mM DCFH-DA for 60 minutes at 37 °C. DMSO and H_2_O_2_ acted as negative and positive controls, respectively.

Two compounds with the most promising bioactivity, Ph_2_Sn(S2MoVa) and Ph_2_Sn(S4MoVa), were selected for further cell apoptosis studies, in order to evaluate the primary damage to biological macromolecules through redox reactions, particularly via formation of ROS [68]. ROS are highly reactive molecules/radicals (O_2_, O_2_^•^, H_2_O_2_, HO^•^) that are continuously generated during aerobic metabolism, but when in excessive amounts, can lead to protein and DNA oxidation, protein cross-linking and cell death. Estimation of the levels of ROS in cell culture is an important step towards better understanding the mechanisms that lead to cell death or disease processes. Figure 7 shows that both the Ph_2_Sn(S2MoVa) and Ph_2_Sn(S4MoVa) stimulated ROS generation. ROS production was measured using the ROS-detecting fluorescent dye DCFH-DA and DMSO was used as a control. Both compounds were able to induce ROS production in RT-112 cell lines after 24 h. The significant increase in ROS generation supports the findings that the compounds have high potential cytotoxicity and suggested that the mechanism of cell death was via the simulation of mitochondrial-initiated events [69].

#### 2.7.4. DNA Interaction Studies

**Figure 8:**
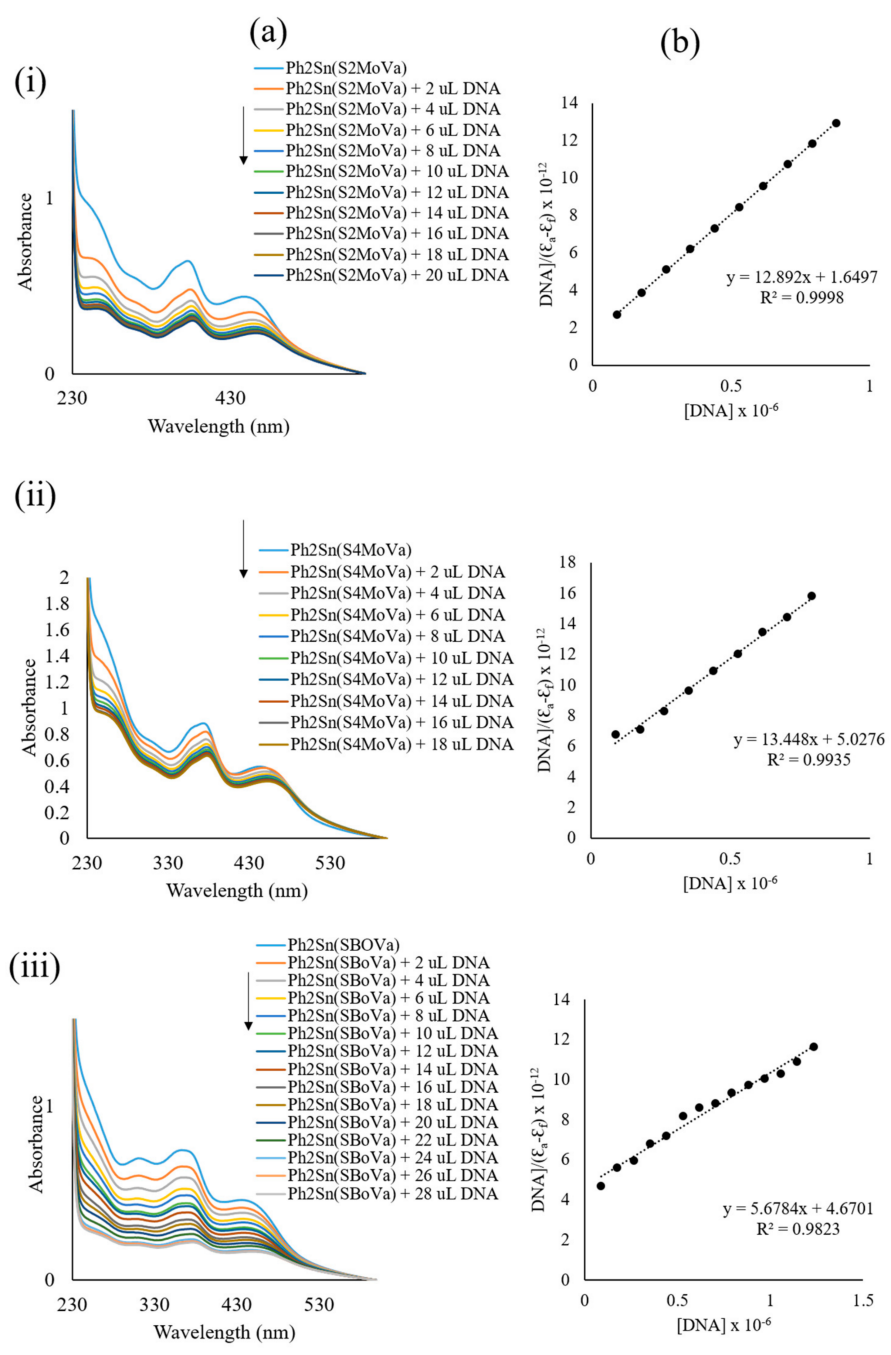
(**a**) Electronic absorption spectra of (**i**) Ph_2_Sn(S2MoVa), (**ii**) Ph_2_Sn(S4MoVa) and (**iii**) Ph_2_Sn(SBoVa); (**b**) Plot of [DNA]/ε_a_ − ε_f_ vs. [DNA] for absorption titration of DNA with (**i**) Ph_2_Sn(S2MoVa), (**ii**) Ph_2_Sn(S4MoVa) and (**iii**) Ph_2_Sn(SBoVa). The arrow indicates the change in absorbance in tandem with increasing DNA concentration.

Both theoretical and experimental DNA binding of the synthesised organotin(IV) compounds with DNA were investigated. The interaction of organotin(IV) compounds with CT-DNA was elucidated via UV-visible absorption spectroscopy. The electronic spectra of the organotin(IV) compounds exhibited three absorption bands around 306–315 nm, 350–377 nm and 433–450 nm, which corresponded to intra-ligand transitions and ligand metal charge transfer (LMCT) transitions (see below). The absorption spectra of diphenyltin(IV) compounds at a constant concentration (50 μM) in the absence and the presence of different concentrations of CT-DNA are shown in Figure 8. The DNA binding spectra for dimethyltin(IV) compounds are presented in Figure S2. Upon increasing concentrations of CT-DNA, hypochromism and bathochromism occurred at around 350–377 nm indicating strong π–π stacking interactions between the aromatic chromophore of the organotin(IV) compounds and the nitrogeneous pair of the DNA strand [11,70]. The same hypochromism and bathochromism shifts were also observed in other transitions. In order to compare the binding strength of the organotin(IV) compounds to CT-DNA, the intrinsic binding constants (K_b_) were quantitatively determined and are presented in Table 3. The K_b_ data showed that the compounds had strong binding affinities, potentially enabling them to block enzyme bound to the nitrogenous bases of DNA.

The Gibbs free energies of interaction between the organotin(IV) compounds and DNA ranged from −31.0 to −37.6 kJ mol^−1^, meaning that the interaction of the organotin(IV) compounds with DNA was spontaneous [71]. To investigate and understand the interactions between DNA and these compounds, molecular docking simulations were carried out.

### 2.8. Molecular Docking Studies

**Figure 9:**
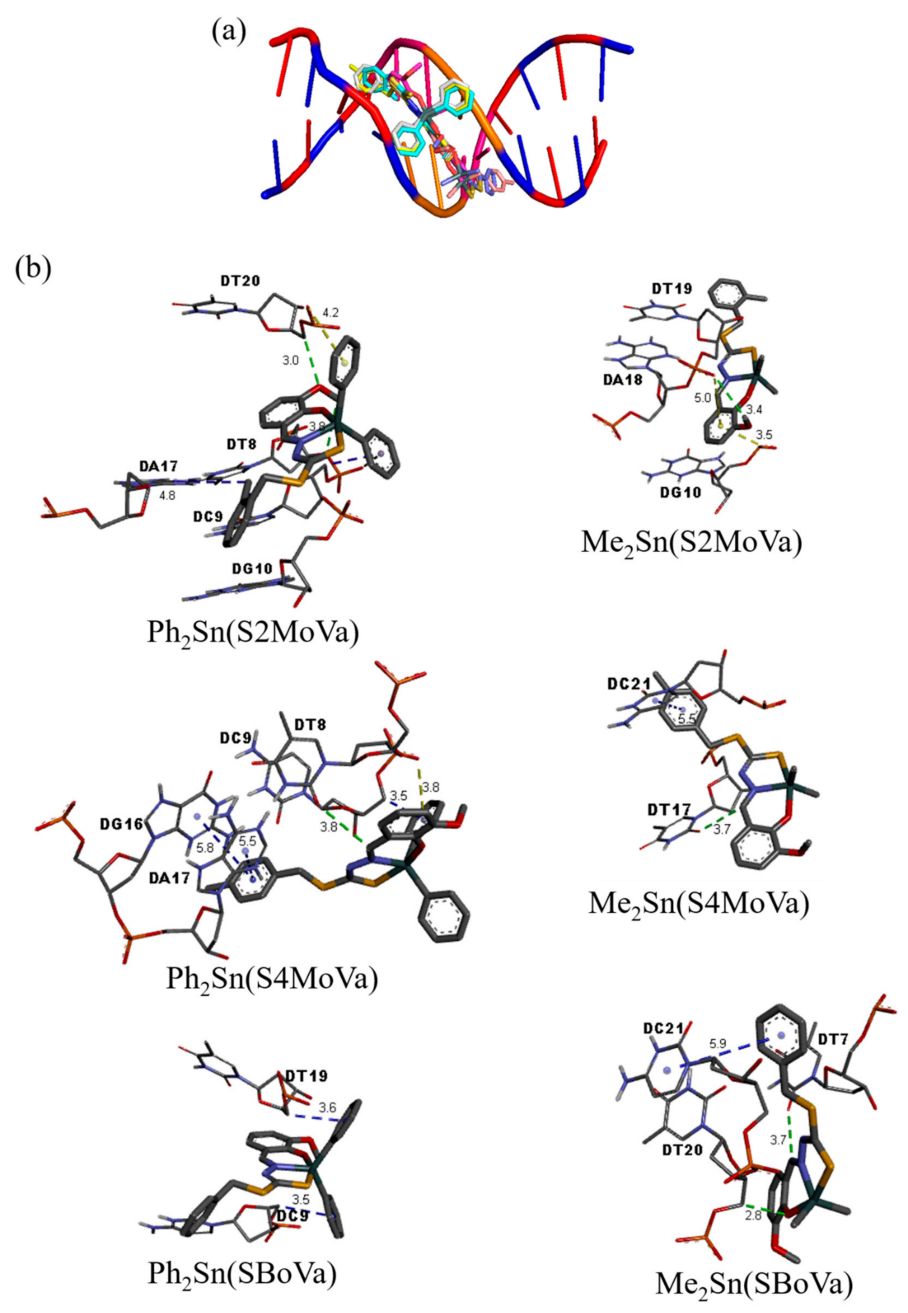
(**a**) Schematic representation of organotin(IV) compounds that fit well in the grooves of the DNA strand obtained by docking simulations. The two double-stranded DNA comprise of the phosphate deoxyribose backbone with guanine (DG, red), cytosine (DC, blue), adenine (DA, pink) and thymine (DT, orange). (**b**) Molecular interactions of organotin(IV) compounds within the grooves of double stranded DNA residues.

Molecular docking analyses of the organotin(IV) compounds with the DNA duplex of sequence d(CGCGAATTCGCG)2 dodecamer (PDB ID: 1BNA) were performed and results are detailed in Table 4. These simulations demonstrated that the organotin(IV) compounds considered here were commensurate with the dimensions of the grooves of the DNA duplex sequence. Figure 9a revealed that these small organotin(IV) compounds fitted well into the A-T rich region of the minor grooves of DNA. Generally, the electronegativity of A-T sequences provided better van der Waals interactions due to their narrower sequences compared to G-C regions. In groove regions, van der Waals and hydrophobic interactions play important roles in the stabilisation of compounds [72]. Their interactions therefore, involved hydrogen bonding, hydrophobic and electrostatic interactions with lowest binding energies between −9.10 to −10.07 kcal mol^−1^, which is better than cisplatin (−8.65 kcal mol^−1^) [25]. The interactions occurred either through the phosphate-oxygen atom of the DNA or via DNA bases. Ph_2_Sn(S2MoVa) exhibited two hydrogen bonding interactions through methoxy-oxygen atoms and methyl groups with phosphate-oxygen atoms of DT20 and DT8, respectively. Similar interactions were observed for Me_2_Sn(S2MoVa) through the oxygen atom of methoxy group with the phosphate backbone atoms of DT19; Ph_2_Sn(S4MoVa), Me_2_Sn(S4MoVa) and Me_2_Sn(SBoVa) interacted through the carbon atoms of the azomethine group with oxygen atoms of thymine base, DT8, DT7 and DT20, respectively. Detailed interactions between the organotin(IV) compounds and DNA residues are presented in Figure 9b. Thus, on the basis of docking studies, it could be inferred that the cytotoxic activities of organotin(IV) compounds could have arisen due to effective interactions of the compounds with DNA.

## 3. Experimental

### 3.1. Physical Measurements

Melting points were determined using an Electrothermal digital melting point apparatus (Cole-Parmer, Staffordshire, UK). IR spectra were recorded using the Perkin Elmer Spectrum 100 with Universal ATR Polarization (PerkinElmer, Boston, MA, USA) in the range 4000–280 cm^−1^. C, H, and N elemental analyses were carried out using a LECO CHNS-932 instrument (LECO, Saint Joseph, MI, USA). Molar conductivities of 10^−3^ M solutions of the organotin(IV) compounds in DMSO were measured at 27 °C using a Jenway 4310 conductivity meter fitted with a dip-type cell with a platinised electrode (Cole-Parmer, Staffordshire, UK). Electronic spectra were recorded on a Shimadzu UV-1650 PC recording spectrophotometer (1000–200 nm) (Shimadzu, Tokyo, Japan). ^1^H and ^13^C NMR spectra were recorded using an NMR JNM ECA400 spectrometer (JEOL, Peabody, MA, USA) with tetramethylsilane (TMS) was used as an internal standard. ^119^Sn NMR were recorded using a JOEL NMR spectrophotometer (JEOL, Peabody, MA, USA). The mass spectra were recorded using a Shimadzu GC-MS QP2010Plus mass spectrometer (Shimadzu, Tokyo, Japan).

### 3.2. Materials

All solvents and reagents were of analytical reagent grade and used without further purification. Chemicals: Hydrazine hydrate, 80% (Fluka, Buchs, Switzerland), benzylchloride, ≥99% (Merck, New York, NY, USA), 2-methybenzyl chloride, 99% (ACROS, Carson, CA, USA), 4-methylbenzyl chloride (ACROS, Carson, CA, USA), potassium hydroxide (HmbG, Hamburg, Germany), carbon disulfide (BDH, Radnor, PA, USA), 2-hydroxy-3-methoxybenzaldehyde (Merck, New York, NY, USA) and nitric acid, 65% (Thermo Fisher Scientific Inc., Waltham, MA, USA), dichlorodiphenyltin(IV) and dichlorodimethyltin(IV) (Sigma Aldrich, St. Louis, MO, USA). Solvents: Acetonitrile (Baker, Sanford, ME, USA), absolute ethanol, 99.8% (Scharlau, Barcelona, Spain), ethanol, 95% (John Kollin Corporation, Midlothian, UK), methanol (Fisher, Pittsburgh, PA, USA) and dimethylsulfoxide (Scharlau, Barcelona, Spain).

Chemicals used for biological assay were purchased as follows: RPMI-1640 medium with l-glutamine (without sodium bicarbonate) (Sigma Aldrich, St. Louis, MO, USA), Penicillin/Streptomycin solution (Biowest, Riverside, MO, USA), Fetal Bovine Serum (sterile filtered) (Biowest, Riverside, MO, USA), Trypsin 0.25%, EDTA in HSSS without calcium, magnesium, with phenol red (Biowest, Riverside, MO, USA), Phosphate Buffered Saline (PBS) 10× concentrate (Sigma Aldrich, St. Louis, MO, USA), dimethyl sulfoxide (DMSO) (Fisher Scientific, Pittsburgh, PA, USA), Annexin-V-FLUOS Staining Kit containing propidium iodide (PI), Annexin-V-Fluorescein, and incubation buffer (ROCHE, Basel, Switzerland). OxiSelect™ Intracellular ROS Assay Kit (Green Fluorescence) containing 20× DCFH-DA, 2,7-dichlorofluorescein (DCF) Standard, Hydrogen Peroxide, and 2× Cell Lysis Buffer (Cell Biolabs, San Diego, CA, USA).

### 3.3. Synthesis

#### 3.3.1. *S*-N-R-Benzyldithiocarbazates (N = 2,3, R = Methyl)

The procedure was adapted from that reported by Tarafder and co-workers [73,74,75]. Potassium hydroxide (11.4 g, 0.2 mol) was dissolved in ethanol (70 mL, 90%). Then, hydrazine hydrate (10 g, 0.2 mol) was added and the mixture was maintained at 0 °C in an ice-salt bath. Carbon disulfide (15.2 g, 0.2 mol) was added dropwise with vigorous stirring (750 rpm) over a period of 1 h. The two layers that formed were separated and the light-brown lower layer was dissolved in 40% ethanol (60 mL) below 5 °C. The mixture was kept in an ice-bath and 2-methylbenzyl chloride/4-methylbenzyl chloride/benzyl chloride (0.2 mol) was added dropwise with vigorous stirring. The sticky white product, which formed, S2MBDTC, S4MBDTC or SBDTC, was filtered and left to dry overnight in a desiccator over anhydrous silica gel. The products were then kept in a freezer.

#### 3.3.2. *S*-2-Methybenzyl-β-*N*-(2-hydroxy-3-methoxybenzylmethylene) Dithiocarbazate (S2MoVaH)

The synthesis of the Schiff base, S2MoVaH was adapted from the literature [76,77]. *S*-2-methylbenzyldithiocarbazate (S2MBDTC) (2.12 g, 10 mmol) was dissolved in hot acetonitrile (100 cm^3^) and added to an equimolar amount of 2-hydroxy-3-methoxybenzaldehyde (1.52 g, 10 mmol) in absolute ethanol (20 cm^3^). The mixture was heated (80 °C) with continuous stirring for about 30 min and then allowed to stand overnight at room temperature. The resultant product was recrystallised from CH_3_CN/EtOH (1:1) to give a light yellow crystalline solid that was filtered and washed with cold absolute ethanol. Yield: 65%. M.p.: 180–183 °C. Elemental analysis: calculated for C_17_H_18_N_2_O_2_S_2_: C, 58.93; H, 5.24; N, 8.09. Found: C, 59.75; H, 5.25; N, 7.81. FT-IR (ATR, cm^−1^): 3084, (N–H); 1600, (C=N); 1117, (N–N); 1026, (C=S). ^1^H NMR (DMSO-*d*_6_) δ (ppm.): 13.34 (s,1H, NH), 9.57 (s, 1H, OH), 8.51 (s, 1H, CH), 6.75–7.34 (multiplet, 7H, Ar–H), 4.40 (s, 2H, CH_2_), 3.76 (s, 3H, O–CH_3_), 2.30 (s, 3H, Bz–CH_3_); ^13^C NMR (DMSO-*d*_6_) δ (ppm.): 196.1 (C=S), 148.6 (C=N); 148.6, 147.4, 144.9, 137.4, 134.4, 130.8, 130.7, 128.2, 126.7, 120.0, 118.8, 114.4 (aromatic-C), 56.4 (O–CH_3_), 36.9 (CH_2_), 19.4 (CH_3_). *m*/*z* calculated for C_17_H_18_N_2_O_2_S_2_: 346.47, found: 346.15.

#### 3.3.3. *S*-4-Methybenzyl-β-*N*-(2-hydroxy-3-methoxybenzylmethylene)dithiocarbazate (S4MoVaH)

The synthesis of the Schiff base, S4MoVaH was similar to S2MoVaH [76,77]. *S*-4-methylbenzyldithiocarbazate (S4MBDTC) (2.12 g, 10 mmol) was dissolved in hot acetonitrile (100 cm^3^) and added to an equimolar amount of 2-hydroxy-3-methoxybenzaldehyde (1.52 g, 10 mmol) in absolute ethanol (20 cm^3^). The mixture was heated (80 °C) with continuous stirring for about 30 min and then allowed to stand overnight at room temperature. The resultant product was recrystallised from CH_3_CN/EtOH (1:1) to give a light yellow crystalline solid that was filtered and washed with cold absolute ethanol. Yield: 68%. M.p.: 177–178 °C. Elemental analysis: Calculated for C_17_H_18_N_2_O_2_S_2_: C, 58.93; H, 5.24; N, 8.09. Found: C, 58.48; H, 5.18; N, 8.76. FT-IR (ATR, cm^−1^): 3092, (N–H); 1598, (C=N); 1118, (N–N); 1030, (C=S). ^1^H NMR (DMSO-*d*_6_) δ (ppm.): 13.32 (s,1H, NH), 9.61 (s, 1H, OH), 8.51 (s, 1H, CH), 6.97–7.24 (multiplet, 7H, Ar–H), 4.39 (s, 2H, CH_2_), 3.76 (s, 3H, O–CH_3_), 2.23 (s,3H, Bz–CH_3_); ^13^C NMR (DMSO-*d*_6_) δ (ppm.): 196.2 (C=S), 148.6 (C=N); 148.6, 147.4, 145.0, 137.0, 134.0, 129.7, 129.6, 120.0, 118.8, 114.4 (aromatic-C), 56.4 (O–CH_3_), 38.0 (CH_2_), 21.2 (CH_3_). *m*/*z* calculated for C_17_H_18_N_2_O_2_S_2_: 346.47, found: 346.10.

#### 3.3.4. *S*-Benzyl-β-*N*-(2-hydroxy-3-methoxybenzylmethylene) Dithiocarbazate (SBoVaH)

The synthesis of Schiff base, SBoVaH was adapted from literature [76,77] *S*-benzyldithiocarbazate (SBDTC) (1.98 g, 10 mmol) was dissolved in hot absolute ethanol (100 cm^3^) and added to an equimolar amount of 2-hydroxy-3-methoxybenzaldehyde (1.52 g, 10 mmol) in absolute ethanol (20 cm^3^). The mixture was heated (78 °C) with continuous stirring for about 30 min and then allowed to stand overnight at room temperature. The resultant product was recrystallised from ethanol to yield light yellow crystals that were filtered and washed with cold absolute ethanol. Yield: 70%. M.p.: 173–174 °C. Elemental analysis: calculated for C_16_H_16_N_2_O_2_S_2_: C, 57.81; H, 4.85; N, 8.43. Found: C, 58.21; H, 4.87; N, 8.28. FT-IR (ATR, cm^−1^): 3089, (N–H); 1598, (C=N); 1125, (N–N); 1030, (C=S). ^1^H NMR (DMSO-*d*_6_) δ (ppm.): 13.34 (s,1H, NH), 9.58 (s, 1H, OH), 8.52 (s, 1H, CH), 6.77-7.37 (multiplet, 8H, Ar–H), 4.45 (s, 2H, CH_2_), 3.77 (s, 3H, O–CH_3_); ^13^C NMR (DMSO-*d*_6_) δ (ppm.): 196.1 (C=S), 148.6 (C=N); 148.6, 147.4, 144.9, 137.3, 129.8, 129.0, 127.8, 120.0, 118.8, 114.4 (aromatic-C), 56.4 (CH_3_), 38.1 (CH_2_). *m*/*z* calculated for C_16_H_16_N_2_O_2_S_2_: 332.44, found: 332.10.

#### 3.3.5. Diphenyltin(IV) Compounds

Each Schiff base (1 mmol) was dissolved in absolute ethanol (50 cm^3^) and mixed with an ethanolic solution (10 cm^3^) of Ph_2_SnCl_2_ (0.34 g, 1 mmol). The resultant yellow solution was refluxed for ca. 6 h. The mixture was left overnight at room temperature and the reduction in the volume of the reaction mixture resulted in the deposition of a yellow precipitate which was filtered off and recrystallised from methanol.

Diphenyltin(IV) [*S*-2-Methybenzyl-β-*N*-(2-hydroxy-3-methoxybenzylmethylene) dithiocarbazate], Ph_2_Sn(S2MoVa)

Yellow solid. Yield: 44%. Melting point: 149–150 °C. Elemental analysis: Calculated for C_29_H_34_N_2_O_6_S_2_Sn: C, 56.42; H, 4.24; N, 4.54. Found: C, 56.52; H, 4.97; N, 4.06. FT-IR (ATR, cm^−1^): 1588, (C=N); 1076, (N–N); 963, (C=S). ^1^H NMR (CDCl_3_) δ (ppm.): 8.77 (s, 1H, CH), 4.47 (s, 2H, CH_2_), 2.43 (s, 3H, CH_3_), 3.97 (s, 3H, O–CH_3_), 6.69-7.94 (multiplet, 17H, Ar–H); ^13^C NMR (CDCl_3_) δ (ppm.): 171.9 (C–S), 166.1 (C=N); 159.3, 152.0, 142.0, 136.1, 130.6, 130.4, 130.2, 128.9, 127.9, 126.3, 126.1, 117.2, 116.2 (aromatic-C), 56.6 (O–CH_3_), 34.4 (CH_2_), 19.4 (Bz–CH_3_).^119^Sn NMR (CDCl_3_) δ (ppm.): −236.38.

Diphenyltin(IV) [*S*-4-Methybenzyl-β-*N*-(2-hydroxy-3-methoxybenzylmethylene) dithiocarbazate], Ph_2_Sn(S4MoVa)

Yellow solid. Yield: 72%. Melting point: 130–132 °C. Elemental analysis calculated for C_17_H_17_N_2_O_2_S_2_: C, 54.82; H, 4.44; N, 4.41. Found: C, 54.42; H, 4.05; N, 4.18. FT-IR (ATR, cm^−1^): 1589, (C=N); 1068, (N–N); 958, (C=S). ^1^H NMR (CDCl_3_) δ (ppm.): 8.74 (s, 1H, CH), 4.41 (s, 2H, CH_2_), 2.32 (s, 3H, CH_3_), 3.94 (s,3H, O–CH_3_), 6.69-7.93 (multiplet, 17H, Ar–H); ^13^C NMR (CDCl_3_) δ (ppm.): 171.8 (C–S), 166.1 (C=N), 159.3, 152.0, 142.0, 137.2, 136.0, 135.8, 133.5, 130.2, 129.4, 129.2, 128.9, 126.1, 117.2, 116.3 (aromatic-C), 56.6 (O–CH_3_), 36.0 (CH_2_), 21.2 (Bz–CH_3_).^119^Sn NMR (CDCl_3_) δ (ppm.): −236.22.

Diphenyltin(IV) [*S*-Benzyl-β-*N*-(2-hydroxy-3-methoxybenzylmethylene) dithiocarbazate], Ph_2_Sn(SBoVa)

Yellow crystals. Yield: 51%. Melting point: 131–133 °C. Elemental analysis calculated for C_28_H_24_N_2_O_2_S_2_Sn: C, 55.74; H, 4.01; N, 4.64. Found: C, 56.35; H, 3.91; N, 4.98. FT-IR (ATR, cm^−1^): 1579, (C=N); 1019, (N–N); 958, (C=S). ^1^H NMR (CDCl_3_) δ (ppm.): 8.74 (s, 1H, CH), 4.45 (s, 2H, CH_2_), 3.96 (s, 3H, O-CH_3_), 6.69-7.93 (multiplet, 18H, Ar–H); ^13^C NMR (CDCl_3_) δ (ppm.): 171.6 (C–S), 166.2 (C=N), 152.0, 141.9, 136.7, 136.1, 130.3, 129.3, 128.9, 128.7, 127.5, 126.1, 117.2, 117.1, 116.2, 115.2, (aromatic-C), 56.6 (O–CH_3_), 36.1 (CH_2_). ^119^Sn NMR (CDCl_3_) δ (ppm.): −236.07.

#### 3.3.6. Dimethyltin(IV) Compounds

Each Schiff base (1 mmol) was dissolved separately in absolute ethanol (50 cm^3^) and dichloromethane (DCM) (20 cm^3^) and then mixed with an ethanolic solution (10 cm^3^) of Me_2_SnCl_2_ (0.22 g, 1 mmol). The resultant yellow solution was refluxed for ca. 6 h. The mixture was left overnight at room temperature and kept for few days yielding brown crystals that were suitable for single crystal X-ray diffraction analysis.

Dimethyltin(IV) [*S*-2-Methybenzyl-β-*N*-(2-hydroxy-3-methoxybenzylmethylene) dithiocarbazate], Me_2_Sn(S2MoVa)

Yellow crystals. Yield: 78%. Melting point: 128–130 °C. Elemental analysis calculated for C_19_H_22_N_2_O_2_S_2_Sn: C, 46.27; H, 4.50; N, 5.68. Found: C, 46.52; H, 4.51; N, 5.82. FT-IR (ATR, cm^−1^): 1580, (C=N); 1076, (N–N); 959, (C=S). ^1^H NMR (CDCl_3_) δ (ppm.): 8.76 (s, 1H, CH), 4.42 (s, 2H, CH_2_), 2.42 (s, 3H, CH_3_), 3.85 (s,3H, O–CH_3_), 6.69–7.34 (multiplet, 7H, Ar–H), 0.97 (Sn–CH_3_); ^13^C NMR (CDCl_3_) δ (ppm.): 173.9 (C–S), 166.3 (C=N), 158.5, 151.5, 137.2, 134.1, 130.6, 130.4, 127.9, 126.3, 126.1, 116.9, 116.5, 115.9 (aromatic-C), 56.3 (CH_3_), 34.6 (CH_2_), 19.4 (Bz–CH_3_), 7.1 (Sn–CH_3_).^119^Sn NMR (CDCl_3_) δ (ppm.): −109.41.

Dimethyltin(IV) [*S*-4-Methybenzyl-β-*N*-(2-hydroxy-3-methoxybenzylmethylene) dithiocarbazate], Me_2_Sn(S4MoVa)

Yellow crystals. Yield: 55%. Melting point: 98–102 °C. Elemental analysis calculated for C_19_H_22_N_2_O_2_S_2_Sn: C, 46.27; H, 4.50; N, 5.68. Found: C, 47.07; H, 4.66; N, 6.11. FT-IR (ATR, cm^−1^): 1589, (C=N); 1071, (N–N); 958, (C=S). ^1^H NMR (CDCl_3_) δ (ppm.): 8.73 (s, 1H, CH), 4.36 (s, 2H, CH_2_), 2.32 (s, 3H, CH_3_), 3.84 (s, 3H, O-CH_3_), 6.69–7.27 (multiplet, 7H, Ar–H), 0.95 (Sn–CH_3_); ^13^C NMR (CDCl_3_) δ (ppm.): 173.7 (C–S), 166.2 (C=N), 158.5, 151.5, 137.1, 133.6, 129.2, 126.1, 119.5, 116.8, 116.4, 115.9 (aromatic-C), 56.3 (CH_3_), 36.2 (CH_2_), 21.2 (Bz–CH_3_), 7.1 (Sn–CH_3_). ^119^Sn NMR (CDCl_3_) δ (ppm.): −109.17.

Dimethyltin(IV) [*S*-Benzyl-β-*N*-(2-hydroxy-3-methoxybenzylmethylene) dithiocarbazate], Me_2_Sn(SBoVa)

Yellow crystals. Yield: 48%. Melting point: 104–106 °C. Analysis calculated for C_18_H_20_N_2_O_2_S_2_Sn: C, 45.11; H, 4.21; N, 5.85. Found: C, 45.36; H, 4.16; N, 6.02. FT-IR (ATR, cm^−1^): 1581, (C=N); 1026, (N–N); 959, (C=S). ^1^H NMR (CDCl_3_) δ (ppm.): 8.73 (s, 1H, CH), 4.40 (s, 2H, CH_2_), 3.85 (s, 3H, O–CH_3_), 6.69-7.40 (multiplet, 8H, Ar–H), 0.95 (Sn–CH_3_); ^13^C NMR (CDCl_3_) δ (ppm.): 173.6 (C–S), 166.3 (C=N), 158.5, 151.5, 136.8, 129.3, 128.7, 127.4, 126.1, 116.9, 116.5, 115.9 (aromatic-C), 56.3 (O–CH_3_), 36.4 (CH_2_), 7.1 (Sn–CH_3_); ^119^Sn NMR (CDCl_3_) δ (ppm.): −108.94.

### 3.4. Single Crystal X-ray Structure Determination

Crystals suitable for single-crystal X-ray diffraction studies were obtained for Me_2_Sn(S2MoVa), Me_2_Sn(S4MoVa) and Me_2_Sn(SBoVa). Unfortunately, suitable crystals for the diphenyltin(IV) compounds were not able to be obtained despite repeated crystal-growth attempts using various techniques and solvent systems. The intensity data for Me_2_Sn(S2MoVa), Me_2_Sn(S4MoVa) and Me_2_Sn(SBoVa) were measured at T = 150 K on an Oxford Diffraction Gemini E CCD diffractometer (Oxford Diffraction Ltd., Agilent Technologies, Santa Clara, CA, USA) fitted with Mo Kα radiation (λ = 0.71073 Å) so that θ_max_ was 29.4°. Data reduction, including analytical absorption correction, was accomplished with CrysAlisPro (Oxford Diffraction Ltd., UK) [78]. The structures were solved by direct methods [79] and refined (anisotropic displacement parameters, C-bound H atoms in the riding model approximation) on *F*^2^. A weighting scheme *w* = 1/[σ^2^(*F*_o_^2^) + (*aP*)^2^ + *bP*] where *P* = (*F*_o_^2^ + 2*F*_c_^2^)/3) [80] was introduced in each case. The crystal of Sn(S2MoVa)Me_2_ was refined as a twin with the fraction of the minor component being 0.1664(20); details are given in the deposited CIF. In the final cycles of the refinement of Me_2_Sn(SBoVa), four reflections, i.e., (−3 4 0), (−1 −1 5), (0 6 0) and (−3 4 4), were omitted owing to poor agreement. The molecular structure diagrams was generated with ORTEP for Windows [81] with 35, 50 and 70% displacement ellipsoids for Me_2_Sn(S2MoVa), Me_2_Sn(S4MoVa) and Me_2_Sn(SBoVa), respectively, and the packing diagrams were drawn with DIAMOND (Crystal Impact, GbR, Bonn, Germany) [82]. Additional data analysis was carried out with PLATON [83]. Crystal data and refinement details are given in Table 5.

### 3.5. Computational Methods

#### 3.5.1. Density Functional Theory (DFT) Calculations

All DFT calculations were performed using Gaussian09 software (Gaussian Inc., Wallingford, CT, USA) [84] and Gaussview5 program (Semichem, Inc., Shawnee Mission, KS, USA) [85] was used to visualise the graphics. The molecular structures and geometries of the Schiff bases and organotin(IV) compounds were fully optimised from the X-ray crystallographic structures using density functional theory (DFT) method with the B3LYP [86,87] hybrid exchange correlation functional with LanL2DZ pseudopotential on Sn [88,89,90] and 6-311G(d,p) Pople basis set for all other atoms. Vibrational frequencies were calculated via the harmonic approximation to ensure that all optimised structured corresponded to local minima of the potential energy surface. The electronic stabilities of the optimised geometries were computed using the time-dependent density functional theory (TD-DFT) formalism [91,92] and included solvation effects (DMSO) via the polarisable continuum method (PCM) [93,94,95], using the same basis set.

#### 3.5.2. Molecular Docking Studies

The coordinates of B-DNA (PDB ID: 1BNA) dodecamer d(CGCGAATTCGCG)_2_ was obtained from the Protein Data Bank (http://www.rcsb.org/pdb). The coordinates of the dimethyltin(IV) compounds, Me_2_Sn(S2MoVa), Me_2_Sn(S4MoVa) and Me_2_Sn(SBoVa) were taken from their crystal structures as a Crystallographic Information File (CIF) and converted to the Protein Data Bank (PDB) format using Mercury software (The Cambridge Crystallographic Data Centre, Cambridge, UK) [96], whereas the coordinates for Ph_2_Sn(S2MoVa), Ph_2_Sn(S4MoVa) and Ph_2_Sn(SBoVa) were obtained after minimisation of energy using DFT method. Molecular docking studies were carried out using the AutoDock Tools version 1.5.6 and the AutoDock version 4.2.5.1 program (Molecular Graphics Laboratory, La Jolla, CA, USA) [97]. Water molecules were removed, polar hydrogen atoms and Kollman charges were added to the receptor (DNA sequences). In the docking analysis, the binding site was assigned across all of the minor and major grooves of the DNA molecule, which was enclosed in a 64 × 64 × 122 grid box with a grid spacing of 0.375 Å. AutoDock was ran using the following parameters: 30 docking trials, population size of 300, maximum number of energy evaluation ranges of 2,500,000, maximum number of generations of 27,000, mutation rate of 0.02, cross-over rate of 0.8 and the default values were used for other parameters. The parameter file of Autodock was modified to incorporate van der Waals interactions and other required parameters for Sn obtained from the Autodock website [98]. The lowest binding energy of the conformation from the highest cluster was selected as the best binding conformation between the ligand and receptor.

### 3.6. Biological Assays

#### 3.6.1. MTT Assays

The inhibitory effect of the synthesised compounds on the growth of bladder (EJ-28 and RT-112) cancer cells was evaluated by MTT assay. The compounds were dissolved in DMSO and diluted in culture medium, where the final concentration of DMSO did not exceed 0.05% (*v*/*v*) and was not toxic to the cancer cells. RT-112 and EJ-28, human bladder cancer cell lines used in this study (ATCC, Manassas, VA, USA) were cultured in RPMI-1640 (High glucose) medium supplemented with 10% fetal bovine serum containing 1% penicillin. The cells were cultured at 37 °C in a humidified atmosphere of 5% CO_2_ in air. Cells were seeded in 96-well plates at 6000 cells per well for 24 h, then they were treated with different concentrations of compounds for 72 h. The medium was subsequently removed and the wells were washed with 200 µL of phosphate buffer saline. Aliquots of 20 µL of 3-(4,5-dimethylthiazol-2-yl)-2,5-diphenyltetrazolium bromide (MTT) was added to each well and incubated for 4 h at 37 °C. Aliquots of 200 µL of media was then removed from each well and 200 µL of DMSO was added. Optical density (OD) was measured using an ELISA plate reader at 570 nm. Control wells (100% viability), containing media and DMSO, were included in all the experiments. All data points represented an average of triplicate assays. Cytotoxicity was expressed as CD_50_, i.e., the concentration that reduced the absorbance of treated cells by 50% with reference to the control (untreated cells) [99].

For HT29 (colon), U87 and SJ-G2 (glioblastoma), MCF-7 (breast), A2780 (ovarian), H460 (lung), A431 (skin), Du145 (prostate), BE2-C (neuroblastoma), MIA (pancreas) cell lines and one normal breast cell line, MCF-10A (normal breast), the MTT assays were performed using the following method:

##### Cell Culture and Stock Solutions

Stock solutions were prepared as follows and stored at −20 °C: Trial compounds were stored as 10 mM solutions in DMSO. All cell lines were cultured in a humidified atmosphere 5% CO_2_ at 37 °C. The cancer cell lines were maintained in Dulbecco’s modified Eagle’s medium (DMEM) (Trace Biosciences) supplemented with 10% foetal bovine serum, 10 mM sodium bicarbonate, penicillin (100 IU/mL), streptomycin (100 μg/mL) and glutamine (4 mM). The normal cell line, MCF-10A was cultured in DMEM:F12 (1:1) cell culture media, 5% heat inactivated horse serum, supplemented with penicillin (50 IU/mL), streptomycin (50 μg/mL), 20mM Hepes, l-glutamine (2 mM), epidermal growth factor (20 ng/mL), hydrocortisone (500 ng/mL), cholera toxin (100 ng/mL) and insulin (10 μg/mL).

##### In Vitro Growth Inhibition Assay

Cells in logarithmic growth were transferred to 96-well plates. Cytotoxicity was determined by plating cells in duplicate in 100 μL medium at a density of 2500–4000 cells/well. On day 0 (24 h after plating), when the cells were in logarithmic growth, 100 μL medium, with or without the test agent, was added to each well. After 72 h drug exposure, growth inhibitory effects were evaluated using the MTT (3-[4,5-dimethylthiazol-2-yl]-2,5-diphenyltetrazolium bromide) assay and absorbance read at 540 nm. Percentage growth inhibition was determined at a fixed drug concentration of 25 μM. A value of 100% was indicative of complete cell growth inhibition. Those analogues showing appreciable percentage growth inhibition underwent further dose-response analysis allowing for the calculation of a GI_50_ value. This value is the drug concentration at which cell growth is 50% inhibited based on the difference between the optical density values on day 0 and those at the end of drug exposure [100,101].

#### 3.6.2. Quantification of Apoptosis by Annexin V

The apoptotic death of RT-112 cells was measured by fluorescence microscopy using Annexin V/PI double-staining. To observe the effect of the diphenyltin(IV) compounds on cell apoptosis, RT-112 cells, which are minimum invasive bladder cancer cells were seeded in 6-well plates at 1 × 10^6^ cells per well. After growth overnight, the inhibition concentration obtained from the CD_50_ analysis was used to treat the cells. After 24 h, the cells were washed with phosphate buffer saline (PBS) and 100 µL of Annexin-V-FLUOS labelling solution containing the incubation buffer and then, propidium iodide (PI) solution was added. The plate was then incubated for 10–15 min at 15–25 °C and analysed by fluorescence microscopy with a Fluorescence Inverted Microscope (Olympus IX51, Waltham, MA, USA). Green staining of the plasma membrane indicated that the cells had bound to Annexin-V. The cells that showed red staining were considered to have lost their membrane integrity. The cells stained green were concluded to be apoptotic cells; the cells with both green and red staining were concluded as late apoptotic cells whereas the cells that showed only red staining were considered as necrotic cells [102].

#### 3.6.3. Measurement of Reactive Oxygen Species (ROS)

Intracellular ROS measurements were used to assess antioxidant or ROS activity. Measurements were performed using a fluorescence plate reader following to the procedure outlines in the ROS detection kit (OxiSelect™). In brief, RT-112 cells were cultured in 100 µL growth medium in a 96-well cell culture plate and incubated in an atmosphere of 5% CO_2_ at 37 °C for a period of 24 h. The growth medium was then removed, the cells were loaded with an oxidation sensitive cell-permeable fluorescent probe, dichloro-dihydro-fluorescein diacetate (DCFH-DA), and subsequently stabilised in the highly reactive DCFH form. In this reactive state, ROS species in living cells were reacted with DCFH, which is rapidly oxidized to the highly fluorescent 2′,7′-dichlorodihydrofluorescein (DCF). The plates were then incubated for 1 h and the growth medium was removed. The cells in each well were washed with PBS and the growth medium containing 3.05 µM of Ph_2_Sn(S4MoVa) compound, negative control (DMSO), or positive control (H_2_O_2_) was added. The plates were incubated in an atmosphere of 5% CO_2_ at 37 °C for a period of 24 h. After incubation, the cells were transferred to black 96-well plates with the addition of 2× Cell Lysis Buffer. The cells were then read on a standard Infinite M200 fluorescence microplate reader (Tecan, Männedorf, Switzerland). The ROS or antioxidant content in unknown samples was determined by comparison with the predetermined DCF standard curve [103].

#### 3.6.4. DNA Binding Studies

DNA binding experiments were carried out at 25°C. DNA concentration per nucleotide was determined using the molar absorption coefficient (6600 M^−1^ cm^−1^) at 260 nm [8,104]. Solutions of calf thymus (CT) DNA in Tris-HCl buffer containing (5 mM Tris, pH 7.2, 50 mM NaCl) [8] showed A_260_/A_280_ of 1.9, indicating that the DNA was sufficiently free of proteins and contaminants [105]. Absorption titration experiments were performed maintaining the concentration of the organotin(IV) compounds solutions at 50 µM and gradually increasing the concentration of CT-DNA. The organotin(IV) compounds were dissolved in DMSO and diluted with Tris-HCI buffer at room temperature (25 °C). The solutions were scanned over 230–600 nm. Absorbance values were recorded 10 min after the addition of DNA solution. The binding constant, K_b_ was determined using the equation:

$$\frac{\left[\mathrm{DNA}\right]}{\left(\varepsilon_{a}-\varepsilon_{f}\right)}=\frac{\left[\mathrm{DNA}\right]}{\left(\varepsilon_{b}-\varepsilon_{f}\right)}+\frac{1}{K_{b}\left(\varepsilon_{b}-\varepsilon_{f}\right)}$$

where [DNA] is the concentration of DNA in base pairs, ε_a_ corresponds to the apparent molar extinction coefficient A_abs_/[M], ε_f_ corresponds to the extinction coefficient for the free organotin [M] and ε_b_ corresponds to the extinction coefficient for the fully bound organotin compound.

## 4. Conclusions

Organotin(IV) compounds derived from S-substituted dithiocarbazates and *o*-vanillin were synthesised and characterised by elemental analysis and various spectroscopic techniques (UV-visible, FTIR, ^1^H, ^13^C and ^119^Sn NMR). The molecular structures of Me_2_Sn(S2MOVa), Me_2_Sn(S4MOVa) and Me_2_Sn(SBOVa) were elucidated by single crystal X-ray structure analysis which indicated that the compounds had five-coordinate C_2_NOS geometries, derived from thiolate-S1, phenoxide-O1 and imine-N atoms of a dinegative, tridentate dithiocarbazate ligand along with two methyl-carbon atoms of the methyl groups, which were intermediate between ideal trigonal-bipyramidal and square-pyramidal. The in vitro cytotoxicity against a panel of cancer cell lines viz., EJ-28 and RT-112 (bladder), HT29 (colon), U87 and SJ-G2 (glioblastoma), MCF-7 (breast), A2780 (ovarian), H460 (lung), A431 (skin), Du145 (prostate), BE2-C, (neuroblastoma) and MIA (pancreas) cancer cell lines revealed the remarkable cytotoxicity of diphenyltin(IV) compounds for most of the cell lines, which were more potent than cisplatin. In order to evaluate the mechanism of death of the organotin(IV) compounds, Annexin-V and ROS assays were performed using the RT-112 cell line where the diphenyltin(IV) compounds were able to induce apoptosis and ROS generation in the cells. The DNA binding of the organotin(IV) compounds were investigated using UV-vis absorption and the diphenyltin(IV) compounds bound reasonably well into the groove of DNA with binding constants in the range 10^5^–10^6^ M^−1^. The mode of interaction of the organotin(IV) compounds with DNA was further validated by molecular docking studies which were in accordance with the experimental results, suggesting the strong binding of diphenyltin(IV) at the AT-rich region of the minor groove of DNA. The observed trend in the cytotoxicity and binding interactions of organotin(IV) compounds were attributed to the nature of the group bound to the Sn(IV) metal centre where the diphenyltin(IV) compounds exhibited potent efficacy and were promising leads for cytotoxic drug development.

## Figures and Tables

**Table 1:** Selected geometric parameters (Å, °) for Me_2_Sn(S2MoVa), Me_2_Sn(S4MoVa) and Me_2_Sn(SBoVa).

Parameter	Me_2_Sn(S2MoVa)			Me_2_Sn(S4MoVa)			Me_2_Sn(SBoVa)		
Bond lengths	molecule a	molecule b	B3LYP	molecule a	molecule b	B3LYP	molecule a	molecule b	B3LYP
			LanL2DZ/6-311G(d,p)			LanL2DZ/6-311G(d,p)			LanL2DZ/6-311G(d,p)
Sn–S1	2.545(2)	2.542(2)	2.576	2.5391(8)	2.5309(7)	2.566	2.5789(11)	2.5607(10)	2.590
Sn–O1	2.092(6)	2.102(6)	2.085	2.090(2)	2.214(2)	2.078	2.103(3)	2.093(3)	2.078
Sn–N2	2.188(6)	2.192(6)	2.285	2.223(2)	2.088(2)	2.232	2.194(3)	2.205(3)	2.226
C1–S1	1.726(9)	1.717(8)	1.751	1.736(3)	1.734(3)	1.753	1.725(4)	1.726(4)	1.748
C1–S2	1.744(8)	1.747(8)	1.772	1.752(3)	1.757(3)	1.771	1.771(4)	1.763(4)	1.783
N1–N2	1.394(9)	1.406(9)	1.389	1.402(3)	1.409(3)	1.388	1.397(5)	1.395(4)	1.383
C1–N1	1.304(11)	1.273(10)	1.293	1.289(4)	1.287(4)	1.293	1.298(5)	1.291(5)	1.296
C(x)–N2	1.308(9)	1.306(9)	1.313	1.309(4)	1.307(4)	1.312	1.311(5)	1.304(5)	1.307
Bond angles									
S1–Sn–O1	157.89(17)	158.25(17)	159.5	158.51(7)	155.88(7)	159.7	155.82(9)	154.24(10)	159.8
S1–Sn–N2	77.35(18)	76.94(18)	77.6	77.33(7)	77.78(6)	77.8	76.70(9)	77.13(8)	77.3
O1–Sn–N2	82.2(2)	82.3(2)	82.0	82.13(9)	82.15(8)	82.2	82.76(11)	82.09(12)	82.4
C18–Sn–C(y)	128.9(5)	128.2(5)	123.9	122.58(14)	123.05(14)	123.9	127.70(18)	124.8(2)	123.7
S1–C1–S2	112.1(5)	111.9(5)	113.4	111.46(17)	111.81(17)	113.4	120.2(2)	120.1(2)	120.8
S1–C1–N1	129.1(6)	128.9(6)	128.2	129.1(2)	129.6(2)	128.2	128.7(3)	128.3(3)	127.7
S2–C1–N1	118.9(7)	119.3(6)	118.4	119.5(2)	118.6(2)	118.4	111.1(3)	111.6(3)	111.5

**Table 2:** Summary of the in vitro cytotoxicity of the Schiff bases and their organotin(IV) compounds in several cell lines, determined by MTT assay and expressed as GI_50_ values with standard errors. (GI_50_ is the concentration at which cell growth is inhibited by 50% over 72 h).

Compounds	Growth Inhibition Concentration, GI_50_ (µM)												
	EJ-28	RT-112	HT29	U87	MCF-7	A2780	H460	A431	Du145	BE2-C	SJ-G2	MIA	MCF10A
S2MoVaH	3.4 ± 0.47	4.6 ± 0.45	3.9 ± 0.71	4.3 ± 0.30	2.7 ± 0.21	3.2 ± 0.15	4.3 ± 0.12	3.8 ± 0.03	4.1 ± 0.09	3.1 ± 0.00	3.0 ± 0.09	4.9 ± 0.37	3.1 ± 0.07
Ph_2_Sn(S2MoVa)	0.35 ± 0.05	0.31 ± 0.05	1.8 ± 0.13	4.4 ± 1.7	1.4 ± 0.12	0.23 ± 0.01	2.8 ± 0.00	2.1 ± 0.09	1.7 ± 0.07	0.47 ± 0.05	0.84 ± 0.09	0.34 ± 0.02	1.2 ± 0.30
Me_2_Sn(S2MoVa)	2.5 ± 0.78	2.4 ± 0.77	2.7 ± 0.10	3.6 ± 0.23	3.1 ± 0.12	3.1 ± 0.06	3.3 ± 0.13	3.0 ± 0.03	3.9 ± 0.03	2.9 ± 0.03	2.5 ± 0.30	3.9 ± 0.07	3.0 ± 0.09
S4MoVaH	3.6 ± 0.44	3.2 ± 0.51	8.0 ± 3.5	3.9 ± 0.00	3.5 ± 0.00	3.1 ± 0.40	5.5 ± 0.73	4.2 ± 0.74	5.4 ± 0.23	3.6 ± 0.27	3.3 ± 0.87	11 ± 2.3	3.5 ± 0.23
Ph_2_Sn(S4MoVa)	3.1 ± 0.37	1.7 ± 0.49	0.87 ± 0.22	1.3 ± 0.40	0.82 ± 0.14	0.35 ± 0.07	1.9 ± 0.23	1.4 ± 0.38	1.5 ± 0.31	0.53 ± 0.01	1.0 ± 0.16	0.39 ± 0.03	1.1 ± 0.37
Me_2_Sn(S4MoVa)	>5.0	>5.0	6.9 ± 3.5	3.3 ± 0.18	3.3 ± 0.32	3.1 ± 0.63	4.7 ± 0.46	4.2 ± 0.34	4.2 ± 0.10	3.3 ± 0.23	3.1 ± 0.55	8.9 ± 1.30	3.3 ± 0.15
SBoVaH	3.1 ± 0.20	3.7 ± 0.57	2.2 ± 0.033	3.1 ± 0.26	2.5 ± 0.12	3.0 ± 0.06	3.3 ± 0.21	3.0 ± 0.15	3.4 ± 0.30	2.8 ± 0.20	2.4 ± 0.26	4.5 ± 0.25	3.0 ± 0.07
Ph_2_Sn(SBoVa)	0.32 ± 0.05	0.58 ± 0.29	1.2 ± 0.22	1.1 ± 0.39	0.90 ± 0.16	0.23 ± 0.03	2.2 ± 0.00	1.6 ± 0.07	1.4 ± 0.33	0.50 ± 0.05	0.74 ± 0.15	0.43 ± 0.04	1.3 ± 0.20
Me_2_Sn(SBoVa)	3.4 ± 0.30	2.4 ± 0.33	2.8 ± 0.15	4.5 ± 0.19	2.5 ± 0.21	3.2 ± 0.03	3.8 ± 0.10	3.2 ± 0.03	3.2 ± 0.13	2.7 ± 0.13	2.1 ± 0.12	5.0 ± 0.09	2.8 ± 0.41
Cisplatin	3.97 ± 0.48	3.56 ± 0.88	11.0 ± 2.0	4.0 ± 1.0	6.5 ± 0.8	1.0 ± 0.1	0.9 ± 0.2	2.4 ± 0.3	1.2 ± 0.1	1.9 ± 0.2	0.4 ± 0.1	8.0 ± 1.0	nd

GI_50_ (μM): (the colors indicate) = 0.1–0.99; = 1.0–9.9; = 10–100; = nd (not determined).

**Table 3:** Binding constants (K_b_), hypochromism (%), bathochromic shifts (nm) and Gibbs free energy (kJ mol^−1^) values for the interaction of organotin(IV) compounds with calf thymus DNA (CT-DNA).

Compounds	Binding Constant (K_b_)	Hypochromism, (%)	Bathochromism Shift (nm)	Gibbs Free Energy (kJ mol^−1^)
Ph_2_Sn(S2MoVa)	6.21 × 10^5^	41.06	6	−33.1
Me_2_Sn(S2MoVa)	3.46 × 10^5^	37.97	10	−31.6
Ph_2_Sn(S4MoVa)	2.67 × 10^5^	28.25	4	−31.0
Me_2_Sn(S4MoVa)	4.15 × 10^5^	43.72	7	−32.1
Ph_2_Sn(SBoVa)	1.21 × 10^6^	70.72	12	−34.7
Me_2_Sn(SBoVa)	3.91 × 10^6^	53.26	1	−37.6

**Table 4:** Molecular docking data for the organotin(IV) compounds with B-DNA (PDB ID: 1BNA) dodecamer d(CGCGAATTCGCG)_2_.

Complex	Final Intermolecular Energy, kcal mol^−1^			Final Total Internal Energy (2), kcal mol^−1^	Torsional Free Energy (3), kcal mol^−1^	Unbound System’s Energy [=(2)] (4), kcal mol^−1^	Estimated Free Energy of Binding [(1) + (2) + (3) − (4)], kcal mol^−1^	Estimated Free Energy of Binding, kJ mol^−1^
	vdW + Hbond + desolv. Energy	Electrostatic Energy	Total (1)					
Ph_2_Sn(S2MoVa)	−11.46	0.00	−11.47	−2.19	1.79	−2.19	−9.68	−40.50
Me_2_Sn(S2MoVa)	−10.27	−0.03	−10.30	0.57	1.19	−0.57	−9.10	−38.07
Ph_2_Sn(S4MoVa)	−11.87	0.01	−11.86	−1.82	1.79	−1.82	−10.07	−42.13
Me_2_Sn(S4MoVa)	−10.84	0.00	−10.83	−0.53	1.19	−0.53	−9.64	−40.33
Ph_2_Sn(SBoVa)	−11.07	0.03	−11.04	−1.86	1.79	−1.86	−9.25	−38.70
Me_2_Sn(SBoVa)	−10.58	−0.01	−10.59	−0.54	1.19	−0.54	−9.40	−39.33

**Table 5:** Crystal data and refinement details for compounds Me_2_Sn(S2MoVa), Me_2_Sn(S4MoVa) and Me_2_Sn(SBoVa).

Compound	Me_2_Sn(S2MoVa)	Me_2_Sn(S4MoVa)	Me_2_Sn(SBoVa)
Formula	C_19_H_22_N_2_O_2_S_2_Sn	C_19_H_22_N_2_O_2_S_2_Sn	C_18_H_20_N_2_O_2_S_2_Sn
Formula weight	493.19	493.19	479.19
Crystal colour	light-yellow	light-yellow	light-yellow
Crystal size/mm^3^	0.03 × 0.05 × 0.10	0.05 × 0.13 × 0.20	0.03 × 0.05 × 0.10
Crystal system	Monoclinic	Triclinic	Triclinic
Space group	*Pn*	*P* $\bar{1}$	*P* $\bar{1}$
*a*/Å	11.3716(9)	10.5908(4)	8.9408(4)
*b*/Å	12.0262(8)	13.9243(5)	10.4182(4)
*c*/Å	16.3644(15)	14.3974(5)	22.6034(12)
*α*/°	90	95.596(3)	97.713(4)
*β*/°	109.285(9)	93.693(3)	94.600(4)
*γ*/°	90	102.116(2)	111.634(4)
*V*/Å^3^	2112.4(3)	2058.11(13)	1920.11(16)
*Z*	4	4	4
*D*_c_/g cm^−3^	1.551	1.592	1.658
*F*(000)	992	992	960
μ(Mo*K*α)/mm^−1^	1.422	1.46	1.562
Measured data	6348	18090	15229
θ range/°	3.6–29.4	3.5–29.4	3.4–29.4
Unique data	6348	9402	8720
Observed data (*I* ≥ 2.0σ(*I*))	5090	7605	6671
No. parameters	478	477	457
*R*, obs. data; all data	0.037; 0.064	0.034; 0.070	0.041; 0.083
*a*; *b* in weighting scheme	0.022; 0	0.026; 0.240	0.032; 0.781
*R*_w_, obs. data; all data	0.055; 0.074	0.049; 0.078	0.062; 0.092
GoF	0.99	1.01	1.01
Range of residual electron			
density peaks/eÅ^−3^	−0.56–0.66	−0.46–0.75	−1.03–0.63

## Supplementary Materials

The following are available online at http://www.mdpi.com/1422-0067/20/4/854/s1. Crystallographic data for Me_2_Sn(S2MoVa), Me_2_Sn(S4MoVa) and Me_2_Sn(SBoVa) reported in this paper have been deposited with the Cambridge Crystallographic Data Centre (CCDC) as supplementary publication nos 1834057-1834059. These data can be obtained free of charge via www.ccdc.cam.ac.uk/getstructures.
