# Supplementary material for: o-Vanillin Derived Schiff Bases and Their Organotin(IV) Compounds: Synthesis, Structural Characterisation, In-Silico Studies and Cytotoxicity
Source: Int J Mol Sci. 2019 Feb 15;20(4):854. doi: 10.3390/ijms20040854 (PMC6413231; doi:10.3390/ijms20040854)
Supplement: Supplementary file 1 [file ijms-20-00854-s001.pdf]

Supplementary: *o*-Vanillin Derived Schiff Bases and Their Organotin(IV) Compounds: Synthesis, Structural Characterisation, In-Silico Studies and Cytotoxicity

Enis Nadia Md Yusof [1,2], Muhammad A. M. Latif [1], Mohamed I. M. Tahir [1], Jennette A. Sakoff [3], Michela I. Simone [2,4], Alister J. Page [2,*], Abhi Veerakumarasivam [5,6], Edward R. T. Tiekink [7] and Thahira B. S. A. Ravoof [1,8,*]

[1] Department of Chemistry, Faculty of Science, Universiti Putra Malaysia, 43400 UPM Serdang, Selangor Darul Ehsan, Malaysia; enisnadia89@gmail.com (E.N.M.Y.); aliflatif@upm.edu.my (M.A.M.L.); ibra@upm.edu.my (M.I.M.T.)

[2] Discipline of Chemistry, School of Environmental and Life Sciences, University of Newcastle, University Drive, Callaghan, NSW 2308, Australia; michela.simone@newcastle.edu.au (M.I.S.); alister.page@newcastle.edu.au (A.J.P.)

[3] Experimental Therapeutics Group, Department of Medical Oncology, Calvary Mater Newcastle Hospital, Edith Street, Waratah NSW 2298, Australia; jennette.sakoff@newcastle.edu.au (J.A.S.)

[4] Priority Research Centre for Chemical Biology & Clinical Pharmacology, University of Newcastle, University Drive, Callaghan, NSW 2308, Australia

[5] Department of Biological Sciences, School of Science and Technology, Sunway University, No. 5 Jalan Universiti, 47500 Bandar Sunway, Selangor Darul Ehsan, Malaysia; abhiv@sunway.edu.my (A.V.)

[6] Medical Genetics Laboratory, Faculty of Medicine and Health Sciences, Universiti Putra Malaysia, 43400 UPM Serdang, Selangor Darul Ehsan, Malaysia

[7] Research Centre for Crystalline Materials, School of Science and Technology, Sunway University, No. 5 Jalan Universiti, 47500 Bandar Sunway, Selangor Darul Ehsan, Malaysia; edward.tiekink@gmail.com (E.R.T.T.)

[8] Materials Synthesis and Characterization Laboratory, Institute of Advanced Technology, Universiti Putra Malaysia, 43400 UPM Serdang, Selangor Darul Ehsan, Malaysia; thahira@upm.edu.my (T.B.S.A.R.)

* Correspondence: alister.page@newcastle.edu.au (A.J.P.); thahira@upm.edu.my (T.B.S.A.R.)

Table S1. Experimental and calculated FTIR vibrations (cm^{-1}) for the Schiff bases and their organotin(IV) compounds.

Compound	Method	IR bands (cm^{-1})				
		v(o-OH)	v(NH)	v(C=N)	v(N-N)	v(C=S)/ v(C-S)
S2MoVaH	Experimental	-	3084	1600	1117	1026
	B3LYP/6-311G(d,p)	3419	3377	1596	1112	1024
Ph_2Sn(S2MoVa)	Experimental	-	-	1588	1076	963
	B3LYP/LanL2DZ/6-311G(d,p)	-	-	1621	1050	968
Me_2Sn(S2MoVa)	Experimental	-	-	1580	1076	959
	B3LYP/LanL2DZ/6-311G(d,p)	-	-	1570	1006	934
S4MoVaH	Experimental	-	3092	1598	1118	1030
	B3LYP/6-311G(d,p)	3415	3378	1596	1112	1024
Ph_2Sn(S4MoVa)	Experimental	-	-	1589	1068	958
	B3LYP/LanL2DZ/6-311G(d,p)	-	-	1625	1047	959
Me_2Sn(S4MoVa)	Experimental	-	-	1589	1071	958
	B3LYP/LanL2DZ/6-311G(d,p)	-	-	1623	1041	966
SBoVaH	Experimental	-	3090	1598	1125	1030
	B3LYP/6-311G(d,p)	3419	3377	1596	1112	1025
Ph_2Sn(SBoVa)	Experimental	-	-	1579	1019	958
	B3LYP/LanL2DZ/6-311G(d,p)	-	-	1624	1048	959
Me_2Sn(SBoVa)	Experimental	-	-	1581	1026	959
	B3LYP/LanL2DZ/6-311G(d,p)	-	-	1619	1047	969

Table S2. ^{1}H NMR spectral data for the Schiff bases and their organotin(IV) compounds.

Compound	^{1}H NMR Assignment, δ (ppm)							
	NH	OH	CH	CH_2	O-CH_3	Ar- CH_3	Sn-CH_3	Aromatic Protons
S2MoVaH	13.34 (s, 1H)	9.57 (s, 1H)	8.51 (s, 1H)	4.40 (s, 2H)	3.76 (s, 3H)	2.30 (s, 3H)	-	6.75-7.34 (m,7H)
Ph_2Sn(S2MoVa)	-	-	8.77 (s, 1H)	4.47 (s, 2H)	3.97 (s, 3H)	2.43 (s, 3H)	-	6.69-7.94 (m,17H)
Me_2Sn(S2MoVa)	-	-	8.76 (s,1H)	4.42 (s, 2H)	3.85 (s, 3H)	2.42 (s, 3H)	0.97 (s, 6H)	6.69-7.34 (m,7H)
S4MoVaH	13.32 (s, 1H)	9.61 (s, 1H)	8.51 (s, 1H)	4.39 (s, 2H)	3.76 (s, 3H)	2.23 (s, 3H)	-	6.97-9.79 (m,7H)
Ph_2Sn(S4MoVa)	-	-	8.74 (s, 1H)	4.41 (s, 2H)	3.94 (s, 3H)	2.32 (s, 3H)	-	6.69-7.93 (m,17H)
Me_2Sn(S4MoVa)	-	-	8.73 (s, 1H)	4.36 (s, 2H)	3.84 (s, 3H)	2.32 (s, 3H)	0.95 (s, 6H)	6.69-7.27 (m,7H)
SBoVaH	13.34 (s, 1H)	9.58 (s, 1H)	8.52 (s, 1H)	4.45 (s, 2H)	3.77 (s, 3H)	-	-	6.77-7.37 (m,8H)
Ph_2Sn(SBoVa)	-	-	8.74 (s, 1H)	4.45 (s, 2H)	3.96 (s, 3H)	-	-	6.69-7.93 (m,18H)
Me_2Sn(SBoVa)	-	-	8.73 (s, 1H)	4.40 (s, 2H)	3.85 (s, 3H)	-	0.95 (s, 6H)	6.69-7.40 (m,8H)

Table S3. $^{13}C\{^1H\}$ NMR spectral data for the Schiff bases and their organotin(IV) compounds.

Compound	Solvent	$^{13}C\{^1H\}$ NMR Assignment, δ (ppm)						
		C=S/C-S	C=N	O-CH_3	CH_2	Ar-CH_3	Sn-CH_3	Aromatic carbons
S2MoVaH	DMSO-d_6	196.1	148.6	56.4	36.9	19.4	-	114.4, 118.8, 120.0, 126.7, 128.2, 130.7, 130.8, 134.4, 137.4, 144.9, 147.4, 148.6
Ph_2Sn(S2MoVa)	$CDCl_3$	171.9	166.2	56.6	34.4	19.4	-	116.2, 117.2, 126.1, 126.3, 127.9, 128.9, 130.2, 130.4, 130.6, 136.1, 142.0, 152.0, 159.3
Me_2Sn(S2MoVa)	$CDCl_3$	173.9	166.3	56.3	34.6	19.4	7.1	115.9, 116.5, 116.9, 126.1, 126.3, 127.9, 130.4, 130.6, 134.1, 137.2, 151.5, 158.5
S4MoVaH	DMSO-d_6	196.2	148.6	56.4	38.0	21.2	-	114.4, 118.8, 120.0, 129.6, 129.7, 134.0, 137.0, 145.0, 147.4, 148.6
Ph_2Sn(S4MoVa)	$CDCl_3$	171.8	166.1	56.6	36.0	21.2	-	116.3, 117.2, 126.1, 128.9, 129.2, 129.4, 130.2, 133.5, 135.8, 136.0, 137.2, 142.0, 152.0, 159.3
Me_2Sn(S4MoVa)	$CDCl_3$	173.7	166.2	56.3	36.2	21.2	7.1	115.9, 116.4, 116.8, 119.5, 126.1, 129.2, 133.6, 137.1, 151.5,158.5
SBoVaH	DMSO-d_6	196.1	148.6	56.4	38.1	-	-	114.4, 118.8, 120.0, 127.8, 129.0, 129.8, 137.3, 144.9, 147.4, 148.6
Ph_2Sn(SBoVa)	$CDCl_3$	171.6	166.2	56.6	36.1	-	-	115.2, 116.2, 117.1, 117.2, 126.1, 127.5, 128.7, 128.9, 129.3, 130.3, 136.1, 136.7, 141.9, 152.0
Me_2Sn(SBoVa)	$CDCl_3$	173.6	166.3	56.3	36.4	-	7.1	115.9, 116.5, 116.9, 126.1, 127.4, 128.7, 129.3, 136.8, 151.5, 158.5

Table S4. Geometric (Å, °) details of the specified intermolecular interactions for $Me_2Sn(S2MoVa)$.

Atoms			Bond lengths (Å)			Bond angle (°)	Direction
C10a	H10a	O1	0.95	2.58	3.490(9)	160	x, -1+y, z
C2a	H2a1	Cg(C11-C16)	0.99	2.78	3.568(10)	137	x, -1+y, z
C2	H2b	Cg(C11a-C16a)	0.99	2.85	3.610(11)	134	x, y, z
C18a	H18f	Cg(C3-C8)	0.98	2.95	3.816(11)	148	-1+x, y, z

Table S5. Geometric (Å, °) details of the intermolecular specified interactions for $Me_2Sn(S4MoVa)$.

Atoms			Bond lengths (Å)			Bond angle (°)	Direction
C8a	H8a	O2a	0.95	2.58	3.341(4)	137	1-x, -y, -z
C17a	H17e	O1a	0.98	2.57	3.526(4)	165	1-x, 1-y, -z
C17a	H17e	O2a	0.98	2.58	3.306(3)	131	1-x, 1-y, -z
C5	H5	Cg(chelate)*	0.95	2.78	3.555(3)	139	1-x, -y, 1-z
C19a	H19e	Cg(C3-C8)	0.98	2.81	3.755(3)	163	1-x, -y, 1-z

*chelate ring defined by Sn1, O1, N2, C10-C12.

Table S6. Geometric (Å, °) details of the intermolecular specified interactions for $Me_2Sn(SBoVa)$.

Atoms			Bond lengths (Å)			Bond angle (°)	Direction
C8a	H8a	Cg(C10a-C15a)	0.95	2.87	3.700(5)	147	-x, -y, 1-z
Cg(chelate)*	Cg(C10-C15)				4.086(2)		-x, 2-y, 2-z
Cg(chelate)**	Cg(C10a-C15a)				4.019(2)		1-x, -y, 1-z

*chelate ring defined by Sn1, S1, N1, N2, C1; closest edge-to-edge contact: N1···C13 = 3.040(6) Å. *chelate ring defined by Sn1a, S1a, N1a, N2a, C1a; closest edge-to-edge contact: N1a···C13a = 3.060(6) Å.

Table S7. Experimental and calculated UV-visible absorption data for the Schiff bases and their organotin(IV) compounds.

Compounds	Wavelength (nm)	
	Experimental	B3LYP/6-311G(d,p) or B3LYP/LanLD2Z/6-311G(d,p)
S2MoVaH	371	377
	344	334
Ph_2Sn(S2MoVa)	444	426
	371	366
	315	304
Me_2Sn(S2MoVa)	444	426
	372	358
	308	320
S4MoVaH	389	378
	348	334
Ph_2Sn(S4MoVa)	443	423
	373	358
	306	312
Me_2Sn(S4MoVa)	447	425
	373	357
	308	313
SBoVaH	371	378
	340	334
Ph_2Sn(SBoVa)	433	423
	364	357
	307	313
Me_2Sn(SBoVa)	443	429
	373	366
	315	307

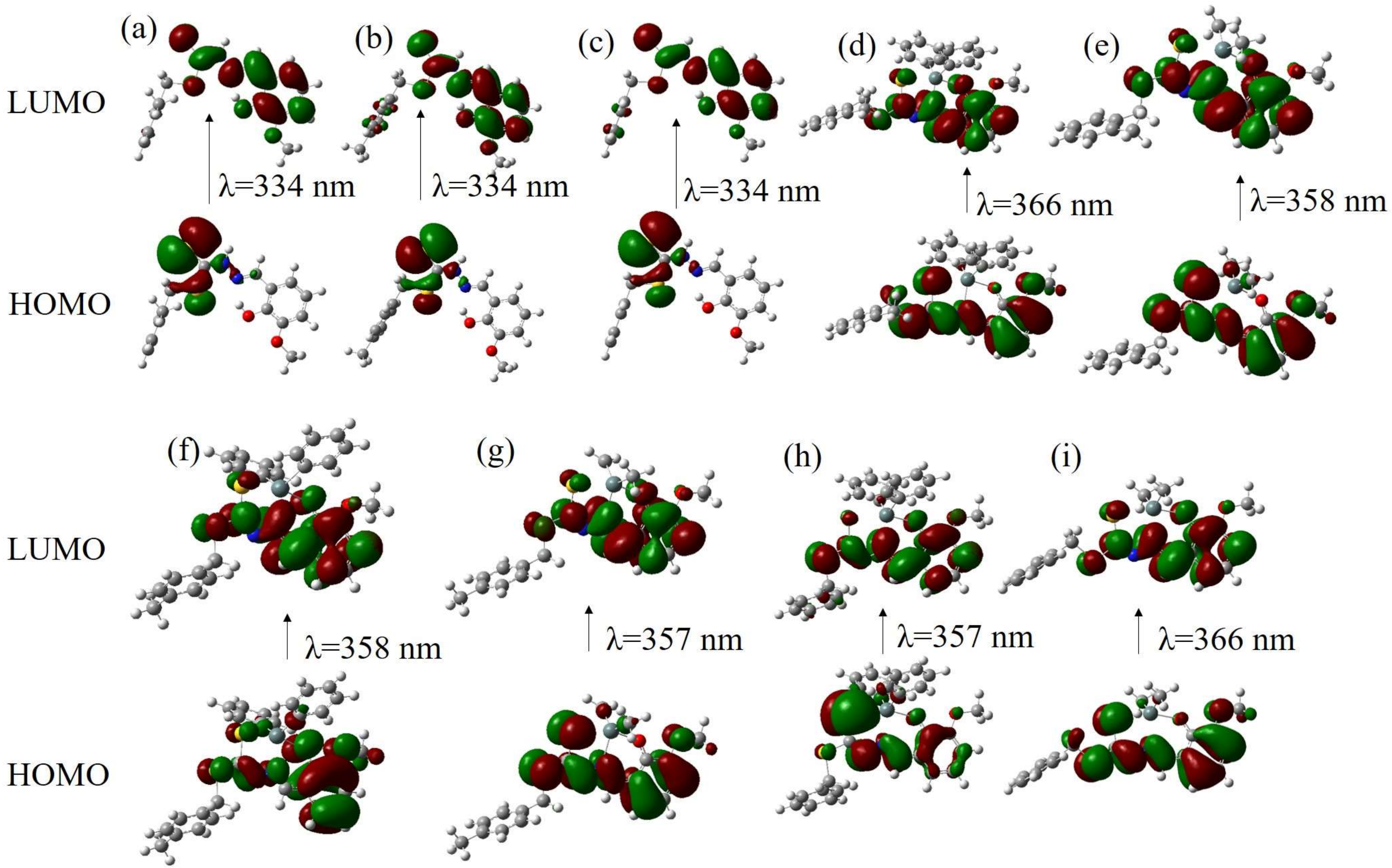

Figure S1. HOMO-LUMO of (a) S2MoVaH, (b) S4MoVaH, (c) SBoVaH, (d) Ph2Sn(S2MoVa), (e) Me2Sn(S2MoVa), (f) Ph2Sn(S4MoVa), (g) Me2Sn(S4MoVa), (h) Ph2Sn(SBoVa) and (i) Me2Sn(SBoVa).

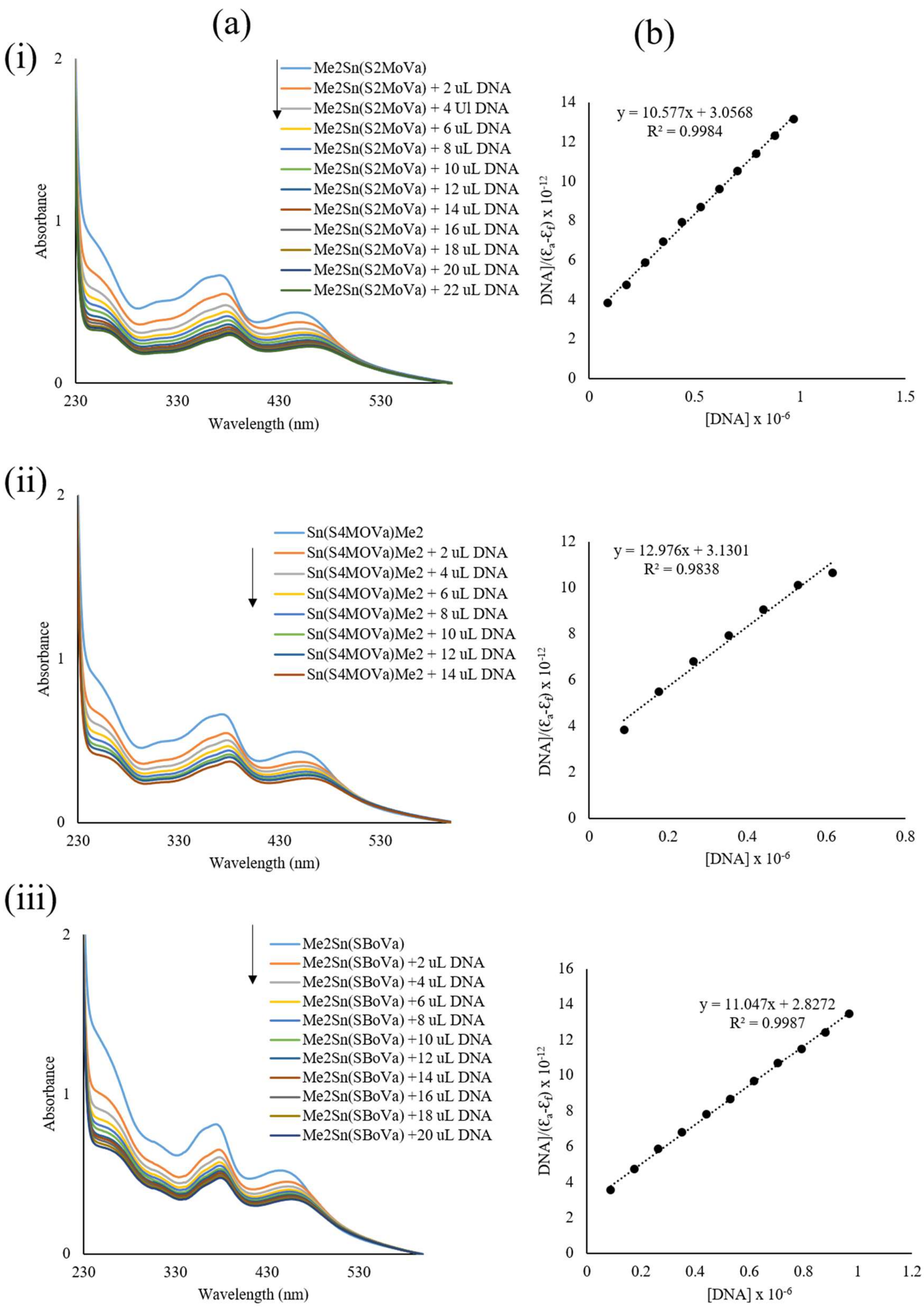

Figure S2. (a) Electronic absorption spectra of (i) Me2Sn(S2MoVa), (ii) Me2Sn(S4MoVa) and (iii) Me2Sn(SBoVa) and (b) Plot of [DNA]/εa - εf vs [DNA] for absorption titration of DNA with (i) Me2Sn(S2MoVa), (ii) Me2Sn(S4MoVa) and (iii) Me2Sn(SBoVa). (The arrow indicates the change in absorbance in tandem with increasing DNA concentration.).
